# Transcriptome-wide analysis of Arabidopsis DICER-LIKE1 RNA substrates

**DOI:** 10.1093/nar/gkaf1434

**Published:** 2026-01-22

**Authors:** Nicolas G Bologna, Alexis Sarazin, Gregory Schott, Antoine Bouet, Natalia P Achkar, Belen Moro, Florence Jay, Uciel Chorostecki, Emanuel A Devers, Olivier Voinnet

**Affiliations:** Department of Biology, Swiss Federal Institute of Technology (ETH), Zürich, 8092, Switzerland; Centre for Research in Agricultural Genomics (CRAG), CSIC-IRTA-UAB-UB, Bellaterra, Barcelona 08193, Spain; Department of Biology, Swiss Federal Institute of Technology (ETH), Zürich, 8092, Switzerland; Department of Biology, Swiss Federal Institute of Technology (ETH), Zürich, 8092, Switzerland; Centre for Research in Agricultural Genomics (CRAG), CSIC-IRTA-UAB-UB, Bellaterra, Barcelona 08193, Spain; Centre for Research in Agricultural Genomics (CRAG), CSIC-IRTA-UAB-UB, Bellaterra, Barcelona 08193, Spain; Centre for Research in Agricultural Genomics (CRAG), CSIC-IRTA-UAB-UB, Bellaterra, Barcelona 08193, Spain; Department of Biology, Swiss Federal Institute of Technology (ETH), Zürich, 8092, Switzerland; Faculty of Medicine and Health Sciences, Universitat Internacional de Catalunya, Sant Cugat del Vallès, Catalunya 08195, Spain; Department of Biology, Swiss Federal Institute of Technology (ETH), Zürich, 8092, Switzerland; Department of Biology, Swiss Federal Institute of Technology (ETH), Zürich, 8092, Switzerland

## Abstract

In plants, DICER-LIKE1 (DCL1) orchestrates microRNA (miRNA) biogenesis by cleaving imperfect stem-loop precursors within primary transcripts (pri-miRNAs). However, the full spectrum of DCL1 RNA substrates remains unexplored. Here, we report transcriptome-wide RNA immunoprecipitation and deep‐sequencing (RIP-Seq) analyses of the Arabidopsis catalytically inactive DCL1 (DCL1ci), designed to bind but not cleave its targets. In inflorescences, DCL1ci-RIP retrieved nearly all evolutionarily conserved *MIRNA* loci and uncovered many hitherto unknown young *MIRNA* loci. Extensive interactions with both pre-miRNA stem-loops and flanking single-stranded regions were detected, suggesting that DCL1 scans pri-miRNAs prior to stem-loop cleavage. Quantitative binding profiles resolved the specific contribution of paralogous *MIRNA* family members in inflorescences, enabling tissue-level discrimination of pri-miRNA engagement. The analysis also identified hundreds of DCL1ci-interacting non-*MIRNA* loci, including protein-coding genes, transposons, and intergenic regions, with many lacking canonical stem-loop structures. We show that DCL1 promotes 24-nt small RNA biogenesis mostly from helitron-derived transcripts via a pathway genetically distinct from RNA-directed DNA methylation. Moreover, we identify a conserved stem-loop in the *DCL1* 5′-UTR suggesting autoregulatory feedback control. Collectively, our study establishes DCLci-RIP as a robust noninvasive approach for profiling DCL substrates, broadens DCL1’s functional landscape, and provides a foundation for dissecting dynamic DCL–RNA interactions across developmental and stress contexts.

## Graphical Abstract

**Graphical Abstract:**
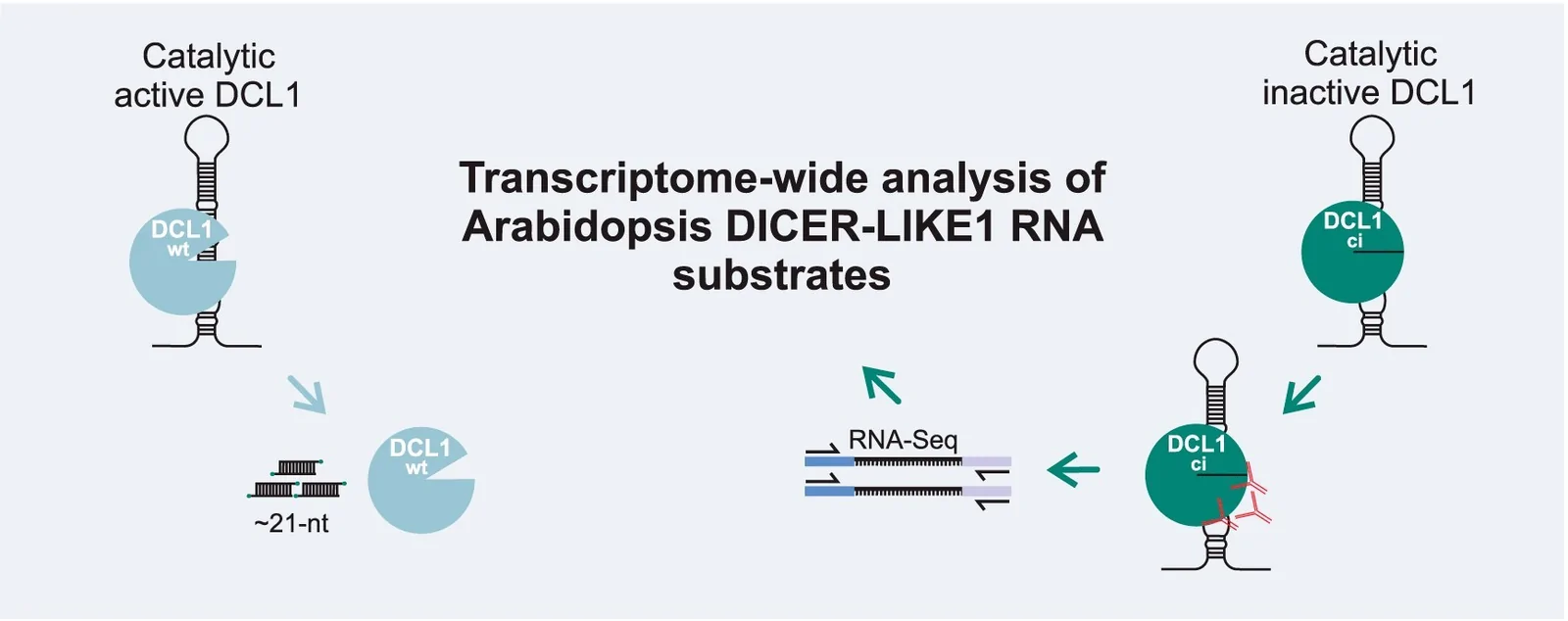

## Introduction

In eukaryotes, RNA silencing controls gene expression via 20–30 nucleotide (nt) small RNAs (sRNAs) that regulate many biological processes including development, stress responses and maintenance of genome integrity [1, 2]. In plants, sRNAs are divided into two main classes depending mostly on the nature of their double-stranded RNA (dsRNA) precursors [3]. MicroRNAs (miRNAs) are produced as single, discrete species from imperfect fold-back structures embedded into endogenous noncoding primary transcripts (pri-miRNAs). miRNAs regulate the levels of fully or partly complementary target transcripts via post-transcriptional gene silencing (PTGS) achieved by endonucleolytic cleavage and/or translational repression coupled to accelerated messenger RNA (mRNA) decay [4]. In contrast, small interfering RNAs (siRNAs) accumulate as populations derived from perfect dsRNA molecules displaying near-perfect complementarity, as produced by e.g. viral replication or endogenous RNA-dependent RNA polymerase (RDR) activities [5]. siRNAs can mediate PTGS or, alternatively, transcriptional gene silencing (TGS) directed on chromatin [6]. Both PTGS and TGS entail the loading of sRNAs into one of several effector proteins in the conserved ARGONAUTE (AGO) family, which, as part of RNA-induced silencing complexes, universally execute silencing of target RNA/DNA [7].

Plant sRNAs derive from their dsRNA precursors by the action of highly conserved Dicer-like (DCL) RNase III-like enzymes organized into modular domains: DExD-box, helicase-C, domain of unknown function 283 (DUF283), PIWI/ARGONAUTE/ZWILLE (PAZ), two RNase III domains, and a dsRNA-binding domain [8, 9]. Of the four DCL proteins encoded in the genome of the model plant *Arabidopsis thaliana*, DCL2, DCL3, and DCL4 process populations of signature 22-, 24-, and 21-nt siRNAs, respectively [10, 11]. DCL3-dependent 24-nt siRNAs originate mostly from transposons and repeats, and mediate TGS via RNA-directed DNA methylation (RdDM) upon their loading into AGO4-clade AGOs [12–15]. Their ∼30–45 bp dsRNA precursors are synthesized predominantly by RDR2 from short single-stranded (ss)RNA templates produced by the plant-specific RNA Polymerase (Pol) IV [16]. DCL4 and its surrogate, DCL2, produce respectively 21- and 22-nt siRNAs from virus-derived dsRNA to execute antiviral silencing predominantly via AGO1 and AGO2 [17, 18]. Generic substrates for the three Arabidopsis siRNA-generating DCLs also include intramolecular fold-back transcripts originating from endogenous inverted repeats (*IRs*; including some evolutionary young miRNA precursors, see below) or from their transgenic counterparts used in experimental RNAi [10, 11]. Recently, a specific role was ascribed to DCL2 and its 22-nt siRNA products in mediating stress adaptation via mostly translational repression [19].

DCL1 produces the majority of miRNAs in Arabidopsis [3]. Once transcribed by RNA polymerase II, capped and polyadenylated, plant pri-miRNAs are processed into precursor miRNAs (pre-miRNAs) by the nuclear microprocessor complex composed of DCL1 and accessory proteins including the dsRNA-binding protein Hyponastic Leaves 1 (HYL1) and the zinc-finger protein Serrate (SE) [20, 21]. DCL1 then operates a second cut on the pre-miRNA to release the mature ∼21bp miRNA/miRNA* duplex. Both 3′ ends of the miRNA duplex are then 2′O-methylated and loaded into mostly AGO1 in the nucleus, although other AGOs including AGO2, AGO7, and AGO10 may also be involved [4]. The AGO1:miRNA complex is then translocated to the cytoplasm in a manner inhibited by leptomycin B, suggesting an EXPORTIN1-dependent pathway [22]. A pool of nonloaded nuclear miRNAs may also reach the cytosol via an EXPORTIN5-mediated pathway, where they may load into neo-translated AGO or, alternatively, move to adjacent cells and over long distances as AGO-free entities [22–24] The microtubule severing enzyme subunit KATANIN1 regulates the extent of cytosolic loading versus movement albeit via unknown mechanisms [25].

According to their conservation and diversification during evolution of the plant kingdom, *MIRNA* genes can be classified as either “ancient” or “young” [26, 27]. Ancient *MIRNA* loci often spawn large paralogous families, of which at least some members are usually highly expressed in a manner underpinned by highly accurate processing by DCL1 [28–30]. Young *MIRNA* loci, by contrast, are single, lowly expressed genes of which the transcription might be enhanced under e.g. stress conditions; their less accurate processing usually depends on DCL1 and/or DCL4 and, hence, is often accompanied by spurious siRNA production [31]. Compared with their stereotypical ∼70-nt animal counterparts, both young and ancient plant pre-miRNAs are variable in length, ranging from 65-nt to over 400-nt, and display a much higher degree of structural heterogeneity [32, 33]. These attributes suggest high flexibility in DCL1’s ability to recognize its RNA substrates. These substrates might encompass other molecules than pri/pre-miRNAs, possibly underlying hitherto unknown, additional functions. What these functions might be has remained an open question, however, because the dsRNA substrates of DCL1, or indeed of any plant DCL, have never been directly isolated. Instead, plant DCLs’ substrates are indirectly inferred by mapping their sequenced products, the sRNAs, to the genomic loci that presumably produce the corresponding dsRNA precursors [11, 31, 34, 35] Previous studies in metazoans using RNA immunoprecipitation (RIP) coupled to deep-sequencing (RIP-Seq) showed that the animal microprocessor proteins DICER, DROSHA, and DGCR8 have a wider spectrum of substrates than originally anticipated [36, 37]. However, these studies employed catalytically active proteins whose substrates were likely incompletely retrieved given the presumably fast cut-and-release action of RNase III proteins upon binding to dsRNA.

**Figure 1.**
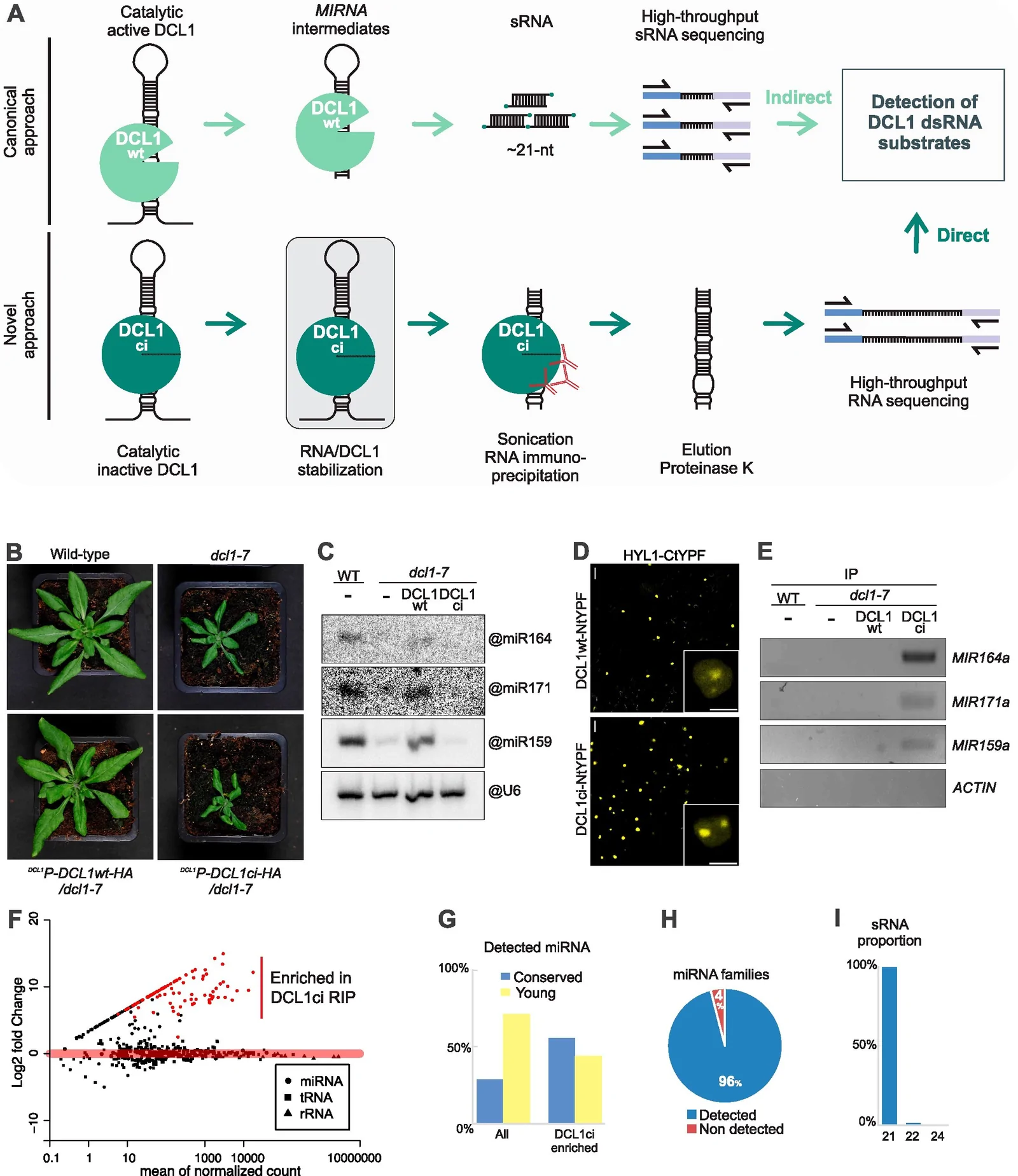
**(A)** Outline of the experimental approach developed for DCL1 RNA substrates’ detection. The canonical approach relies on high-throughput sRNA seq followed by genomic positions’ mapping (upper panel). The novel approach involves DCL1/RNA complexes immunoprecipitation (IP) from plants expressing catalytically inactive (ci) DCL1 followed by high-throughput RNA seq and genomic positions’ mapping (lower panel). **(B)** Phenotypes of wild-type and *dcl1-7* plants expressing wild-type (wt) or catalytically inactive (ci) genomic DCL1 under the *DCL1* endogenous promoter fused to Human influenza hemagglutinin (HA). **(C)** Northern blot analysis of miRNAs levels in *^DCL1^P-DCL1wt-HA/dcl1-7, ^DCL1^P-DCL1ci-HA/dcl1-7*, Wild-type (WT), and *dcl1-7* control plants. U6 is used as a loading control. **(D)** Transient BiFC assays in *Nicotiana benthamiana* leaves used to test interaction between DCL1ci (*35S:*DCL1ci-NtYFP) and HYL1 (*35S:*HYL1-CtYFP). DCL1wt (*35S:DCL1wt-NtYFP*) was used as a positive control and PRMT5 (*35S:PRMT5-NtYFP*) was used as a negative control (see Supplementary Fig. S1E) [39]. **(E)** Validation of DCL1ci substrates by RIP followed by semi-quantitative reverse transcription (sqRT)-PCR of transcript from *MIR159, MIR171, MIR164*, and *ACTIN* in IP fractions of RIPs from *^DCL1^P-DCL1ci-HA/dcl1-7, ^DCL1^P-DCL1wt-HA/dcl1-7*, and control plants. ACTIN is used as a loading control. **(F)** MAplot representation of the differential analysis of reads count from *^DCL1^P-DCL1ci-HA/dcl1-7* and *dcl1-7* control plants (three replicates for each) for miRNAs (circles), transfer RNAs (tRNAs; squares), and ribosomal RNAs (rRNAs; triangles). Loci with an adjusted *P*-value <.05 are indicated in red. **(G)** Proportion of evolutionarily conserved miRNAs (blue) and evolutionarily young miRNAs (yellow) detected in DCL1ci-RIP. **(H)** Fraction of evolutionarily conserved *MIRNA* families identified via DCL1ci-RIP. **(I)** Proportion of 20/21-, 22-, and 24-nt sRNA reads spawn from evolutionarily conserved *MIRNAs* recovered from DCL1ci-RIP seq analysis.

To unbiasedly and exhaustively explore the identity of Arabidopsis DCL1 substrates, we engineered point-mutations in the RNase III domains to produce a catalytically inactive allele of DCL1 (DCL1ci). The mutations were predicted to biochemically stall DCL1ci on its substrates [38], thereby likely optimizing their purification and identification (Fig. 1A). Here, we report the results of a transcriptome-wide RIP-Seq analysis of DCL1 substrates performed in inflorescences of DCL1ci/*dcl1-7* mutant Arabidopsis. The outcomes improve our understanding of the mechanisms, evolution, and genetic diversification of the plant miRNA pathway. They also uncover a variety of hitherto unknown DCL1-bound RNA species suggesting its functional involvement beyond miRNA biogenesis, including at the nexus of several RNA silencing pathways.

## Materials and methods

### Plant material and growth conditions

*A. thaliana* ecotype Col-0 was used as the wild-type in all experiments and is also the genetic background of the previously described *dcl1-11, hyl1-2, hen1-6, dcl2-1/dcl3-1/dcl4-2, rdr2-1, rdr6-15, rdr1-1/rdr2-1/rdr6-15, pol IV (nrpd1a), pol V (nrpd1b), ago1-27, ago2-1*, and *ago4-5*. Seedlings were grown on ½ Murashige and Skoog (MS) medium (pH 5.7) in growth cabinets with an average light intensity of 110–120 µmol m^−2^ s^−1^ and 50% relative humidity. Expression analyses were performed 10-to-14 days post-germination. For inflorescence and leaf tissues, plants were grown in soil in 16-h-light/8-h-dark regimen. Growth chambers were equipped with 36 W fluorescent lights in a 2:1 ratio of 840 Cool White:Grolux with an average light intensity of 110–120 µmol m^−2^ s^−1^ and 50% relative humidity. Stable transgenic plants were produced through the floral dip method [40]. Single-loci homozygous lines were selected via segregation based on antibiotic resistance. A minimum of three lines obtained in independent transformation events were analyzed for each construct to ensure consistent and unbiased results. Transient expression in *N. benthamiana* was performed according to de Felippes *et al.* [41].

### Plasmids, cloning, and mutagenesis

All constructs were created in the pB/K7m34GW vector by multisite Gateway recombination, as described by [42]. Briefly, promoters and coding sequences were amplified from Arabidopsis genomic DNA with Phusion polymerase (Thermo Scientific) using primers listed in Supplementary Table S8 and recombined into the appropriate pDONR plasmid with Gateway BP Clonase (Invitrogen). For DCL1 mutagenesis, point mutations were introduced by amplifying three fragments using primers P1fw/P1rev, P2fw/P2rev, and P3fw/P3rev, respectively. Then polymerase chain reaction (PCR) products were purified and used as templates and amplified using nested P1fw and P3rev primers. Resulting mutated RNase IIIa/b domains were introduced into the entry clones by replacing the WT domains using EcoRV restriction digest and ligation. Fluorescent reporters were similarly amplified and all plasmids sequenced. Three-way recombination was subsequently performed with Gateway LR Clonase to create the promoter:reporter-gene constructs used in this study.

### DCL1 RIP

All steps were performed at 4°C and on ice. Arabidopsis seedlings or inflorescence tissue from the same pooled transgenic lines were collected and cross-linked by vacuum infiltration in ice-cold cross-linking buffer containing 1% (v/v) formaldehyde prepared in sample buffer [10 mM Tris–HCl, pH 7.5, 10 mM Na_2_ ethylenediaminetetraacetic acid (EDTA), 100 mM NaCl, and 0.1% (v/v) Triton X-100; Triton X-100 added post-autoclaving]. Approximately 100 seedlings/inflorescences were immersed in ∼50 ml of cross-linking buffer and subjected to vacuum infiltration at 0.2–0.3 atm (i.e. 0.7–0.8 atm below) for 7 min, followed by slow vacuum release. This step was repeated once to ensure efficient tissue penetration. Cross-linking was quenched by adding glycine to a final concentration of 0.125 M, followed by an additional 7 min vacuum infiltration at 0.2–0.3 atm. Samples were then washed with sample buffer thoroughly, gently dried on *Whatman* paper, frozen in liquid nitrogen, and stored at −80°C until further processing. Frozen tissues (∼500 µl) were ground in liquid nitrogen and lysed in RIP buffer containing 50 mM Tris–HCl, pH 7.5, 150 mM NaCl, 10% (v/v) glycerol, 0.1% (v/v) NP-40, 1 mM EDTA, and 4 mM MgCl_2_. The RIP buffer was sterilized by filtration through a 0.22 µm filter to minimize the risk of introducing RNases from microbial contaminants. Before use, the buffer was supplemented with 1 × cOmplete Mini EDTA-free protease inhibitor cocktail (Roche) and RNase inhibitor (40 U/ml). The same procedure was applied to the N1 and N2 washing buffers before use. Lysates were incubated at 4°C on a rotating wheel for 45 min, filtered through a 40 µm mesh, and sonicated (Bioruptor; high power, 30 s on/30 s off, 5 cycles on ice). Lysates were cleared by two rounds of centrifugation (12 000 × *g*, 10 min, 4°C), and protein concentrations were determined using the Bradford assay. Extracts were normalized across samples, with 20% retained as input and the remaining 80% used for IP. To perform the IP, HA magnetic beads (Thermo Fisher) were pre-washed three times with RIP buffer for 5 min each at 4°C on a rotating wheel. The same procedure was used to pre-washed Dynabeads Protein G (Thermo Fisher). Following washing, HA beads were incubated with blocking solution containing a final concentration of 1 mg/ml of bovine serum albumin (BSA) and 1 mg/ml of *Escherichia coli* transfer RNA (tRNA) for at least 1 h at 4°C on a rotating wheel. In parallel, 20 µl of pre-washed Dynabeads Protein G were added to each IP lysate, and samples were incubated for at least 1 h at 4°C to pre-clear the lysate. After incubation, the lysates were transferred to fresh tubes, and 20 µl of blocked HA beads were added to each sample. The mixtures were incubated for 3 h at 4°C on a rotating wheel to allow IP. Following incubation, HA magnetic beads were washed three times with 1 ml of N1 buffer [50 mM Tris–HCl, pH 7.5, 150 mM NaCl, 10% (v/v) glycerol, 0.1% (v/v) sodium dodecyl sulphate (SDS), 1% (v/v) Triton X-100, 1 mM EDTA, 4 mM MgCl_2_, supplemented with protease inhibitors], each for 15 min at 4°C on a rotating wheel. Beads were then subjected to two additional washes with 1 ml of N2 buffer [50 mM Tris–HCl, pH 7.5, 500 mM NaCl, 10% (v/v) glycerol, 0.1% (v/v) SDS, 1% (v/v) Triton X-100, 1 mM EDTA, 4 mM MgCl_2_, supplemented with protease inhibitors] under the same conditions. After washing, beads were resuspended in RIP. An aliquot representing 25% of the input and immunoprecipitated fractions was set aside for protein analysis, while the remaining 75% was processed for RNA extraction. For protein analysis, *Laemmli* buffer was added, and samples were heated at 95°C for 5 min for the input fraction and 10 min for the IP fraction before storage at –20°C. For RNA extraction, samples were first treated with proteinase K (20 µg) and 6 µl of NaCl at 50°C for 40 min to digest proteins, followed by incubation at 65°C for 30 min to reverse formaldehyde cross-links. RNA was extracted using phenol/chloroform/isoamyl alcohol, followed by ethanol precipitation with glycogen as carrier. RNA pellets were washed twice with 80% ethanol, dried, and resuspended in nuclease-free water (20 µl for input, 10 µl for IP). RNA integrity was assessed using the Agilent RNA 6000 Pico Kit (Agilent Technologies.

### IP of AGO proteins from Arabidopsis inflorescences

Approximately 125 mg of *A. thaliana* Col-0 inflorescence tissue was flash-frozen in liquid nitrogen and ground to a fine powder using a pre-chilled mortar and pestle. The powdered tissue was resuspended in 1 ml of ice-cold lysis buffer containing 50 mM Tris–HCl (pH 7.5), 150 mM NaCl, 10% (v/v) glycerol, 0.1% (v/v) Nonidet *P*-40, protease inhibitor cocktail (Roche), and MG132 (1:10 000 dilution). The lysate was incubated for 30 min at 4°C with gentle rotation. Following incubation, the lysate was centrifuged at 10 000 × *g* for 30 min at 4°C, and the supernatant was transferred to a new microcentrifuge tube. To reduce nonspecific binding, 20 µl of Protein A agarose beads (Roche, Cat. No. 11134515001), pre-equilibrated in lysis buffer, were added to the supernatant for pre-clearing. The mixture was incubated for 30 min at 4°C with gentle rotation, then centrifuged at 5000 × *g* for 1 min at 4°C. The resulting supernatant was transferred to a fresh tube. For IP, specific antibodies were added to the pre-cleared lysate at the following dilutions: A4 (1:400), A6 (1:200), and A9 (1:200). The samples were incubated for 2 h at 4°C with gentle rotation. Subsequently, 40 µl of Protein A agarose beads were added, and the mixture was incubated for an additional 2 h at 4°C with gentle rotation. After incubation, the beads were washed three times with lysis buffer to remove unbound proteins. The immunoprecipitated complexes were then subjected to phenol:chloroform:isoamyl alcohol (25:24:1) extraction. For downstream analyses, 20% of the bead volume was used for protein analysis, while the remaining 80% was allocated for RNA analysis. Proteins were extracted by adding 50 µl of 1× western blot loading buffer directly to the beads, followed by incubation at 95°C for 5 min. RNA was extracted from the remaining beads using TRIzol reagent (Invitrogen), according to the manufacturer’s instructions.

### RNA gel blot analysis

Total RNA was extracted from frozen tissue and ground with pestle and mortar in TRIzol (Invitrogen) following the manufacturer’s instructions. Total RNA and RNA from IPs were separated on 17.5% polyacrylamide-urea gels, electro-transferred to a Hybond-NX membrane (GE Healthcare), and cross-linked via 1-ethyl-3-(3-dimethylaminopropyl) carbodiimide-mediated chemical cross-linking. Oligonucleotides complementary to tasiRNA255 (tasi1), miR159, miR164, miR171, miR822, siRNA Rep2, and U6 (Supplementary Table S8) were end-labeled by incubation with T4 PNK (Thermo Scientific) in the presence of [γ-32P]ATP. PCR probes complementary to *IR71.1, IR71.2, IR71.3, IR71.4, AT1TE37610, AT3TE58810, AT5TE57705, AT3TE61120, AT1TE45720, AT1TE55560, AT4TE22770*, and *AT2TE22890* were used using Prime-a-Gene Labeling System (Promega, Ref. U1100) according to the manufacturer’s instructions. One hundred nanograms of DNA were diluted in nuclease-free water to a total volume of 14.5 μl, denatured at 95°C for 5 min, and immediately cooled on ice for 5 min. The labeling mix was then assembled by adding 18 μl of nuclease-free water, 10 μl of 5 × reaction buffer, 2 μl of BSA, 2 μl of a 0.5 mM mix of dATP, dTTP, and dGTP, 2.5 μl of [α-^32P]-dCTP (10 mCi/ml), and 1 μl of Klenow fragment (DNA Polymerase I, large fragment). The reaction was incubated at 37°C for 1 h and 30 min. Labeled probes were purified using G-50 spin columns (GE Healthcare) to remove unincorporated nucleotides. Finally, probes were denatured again at 95°C for 5 min and chilled on ice for 5 min before use in hybridization experiments. Multiple small RNAs were hybridized on individual membranes by stripping three times with boiling 0.1% SDS and re-probing. Results shown are representative of at least three independent experiments.

### RNA sequencing

RNA immunoprecipitated from inflorescences from three independent pools of pDCL1:DCL1ci-HA/*dcl1-7* and three independent pools of *dcl1-7* were processed into sequencing libraries and sequenced by Fasteris (http://www.fasteris.com; Switzerland) using Illumina HiSeq sequencer. Data are available on the NCBI Gene Expression Omnibus (GEO) under the accession number GSE192355.

### Bioinformatics analysis

#### RNA-seq data processing

Adapter sequences were removed from raw reads using fastx_clipper (options: -c -l 25 -Q 33 -a TGGAATTCTCGG) and aligned against *A. thaliana*’s genome (TAIR10) using Tophat (v2.0.11; options: -I 4000 -p 20 –library-type fr-firststrand) [43]. Coverage files (bigwig) were generated using bamCoverage from deepTools [44] with option –binSize 1 for visualization using IGV [45]. 

**Figure 2.**
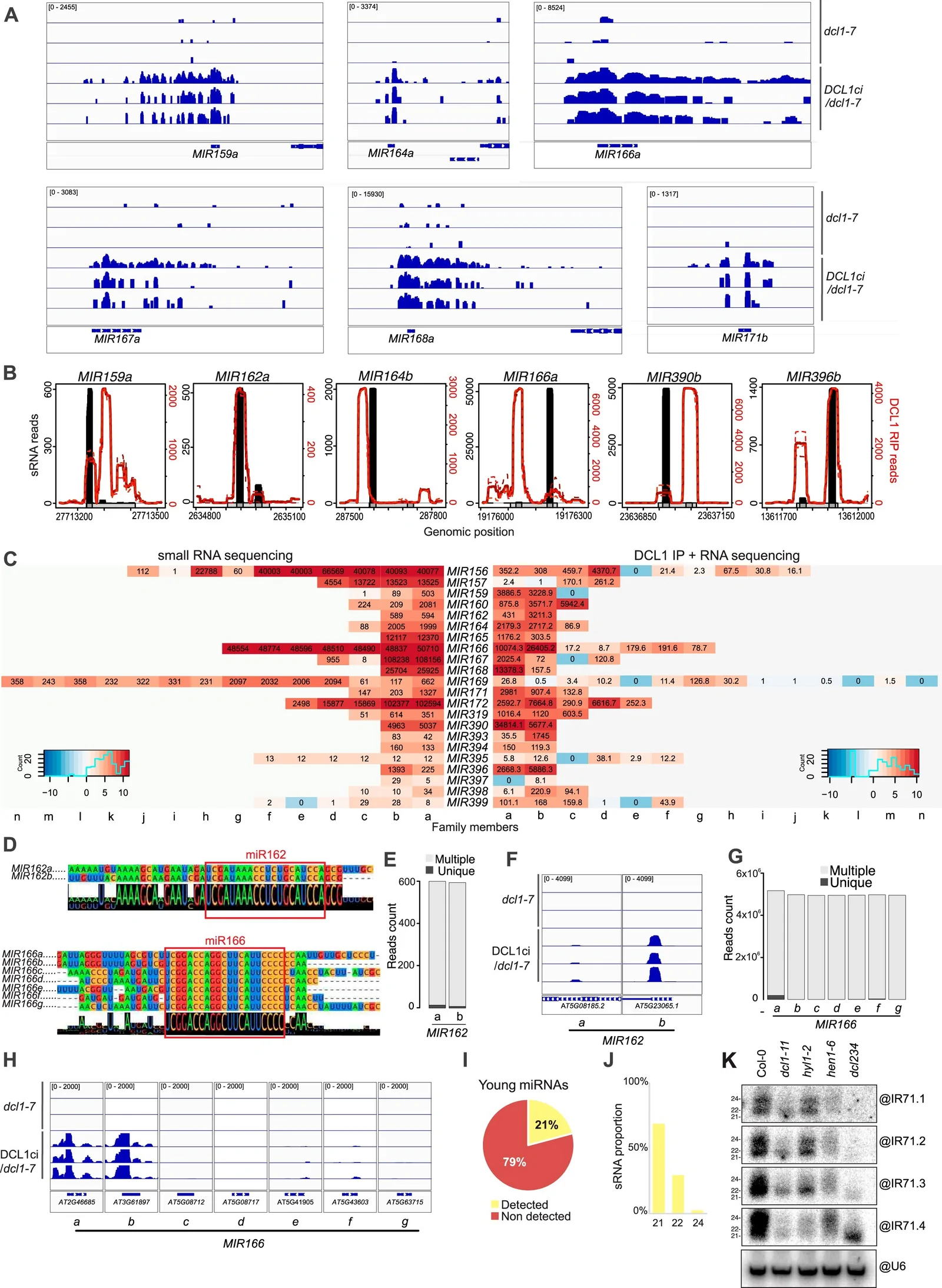
**(A)** Integrative Genomics Viewer (IGV) visualization of reads coverage from three replicates of *^DCL1^P-DCL1ci-HA/dcl1-7* or *dcl1-7* control plants over the *MIR159a, MIR164a, MIR166A, MIR167A, MIR168A*, and *MIR171b* genomic loci and surrounding regions. Visualizations are done using the Log Group Autoscale options with maximum coverage indicated on the first track. **(B)** Coverage of sRNA annotations (black histogram) versus actual RNA seq reads from DCL1ci-RIP (red line) over *MIR159a, MIR162a, MIR164b, MIR166a, MIR390b*, and *MIR396b* precursors. The plain thick red lines correspond to the average of three replicates represented with dashed thin red lines. Light and dark gray rectangles represent primary transcripts and mature 5p/3p miRNA annotations, respectively. **(C)** Heatmap representation of sRNA sequencing reads (left) and DCL1ci-RIP data (right) for evolutionary conserved *MIRNA* families in inflorescences. **(D)** Multiple-alignment of stem-loop sequences from *MIR162* and *MIR166* family members. Red squares indicate mature miRNAs. The full alignments are presented in Supplementary Fig. S2B. **(E)** Reads count from miR162 paralogs a and b in wild-type sRNA sequencing data. Number of reads with a single or multiple genomic positions are indicated in dark and light gray, respectively. **(F)** IGV visualization of reads coverage from three replicates of *^DCL1^P-DCL1ci-HA/dcl1-*7 versus *dcl1-7* control plants over the *MIR162a* loci. **(G)** Same as in panel (E) for the *miR166a-g* paralogs. **(H)** Same as in panel (F) for the seven *MIR166* loci. **(I)** Fraction of evolutionarily young miRNAs families detected via DCL1ci-RIP. **(J)** Proportion of 20–21-, 22-, and 24-nt sRNA reads from young miRNAs recovered from the DCL1ci-RIP seq analysis. **(K)** Northern blot analysis of sRNA accumulation at the DCL1ci-binding sites identified on *IR71* in Col-0 (wild-type), *dcl1-11, hyl1-2, hen1-6*, and *dcl234* (*dcl2-1/dcl3-1/dcl4-2)* backgrounds. The membrane was stripped and re-probed multiple times.

Reads overlapping miRNA stemloop annotations (miRBaseV21) as well tRNA and rRNA annotations (TAIR10) enlarged by 100-nt up and downstream were counted using intersectBed from bedtools [46]. DEseq2 [47] was then used for differential analysis and MAplot representation. Loci with an adjusted *P*-value <.05 were considered as differentially represented between conditions. The relative coverage profile presented in Fig. 1G was generated using R after retrieving the reads covering all miRNA stemloop annotations (miRbase V21) and the 500 bp regions up/downstream with intersectBed and calculating their relative position. For the miRNA specific stem-loop profiles presented in Fig. 2D, whole genome single nucleotide coverage was generated with genomecov from bedtools and then, data for the regions of interest were extracted and use graphical representation using R.

Genome wide identification of DCL1ci enriched regions was done by dividing *A. thaliana* genome into 100 bp windows overlapping by 50 bp. The number of reads overlapping each window were define using intersectBed. Differential analysis was done using DEseq2, only windows with at least one reads in at least one replicates were considered for normalization. Windows with an adjusted *P*-value <.05 and a DCL1ci-IP average normalized reads count of at least 50 were considered as having a significant enrichment. Those were then merged if distant by <200 bp.

**Figure 3.**
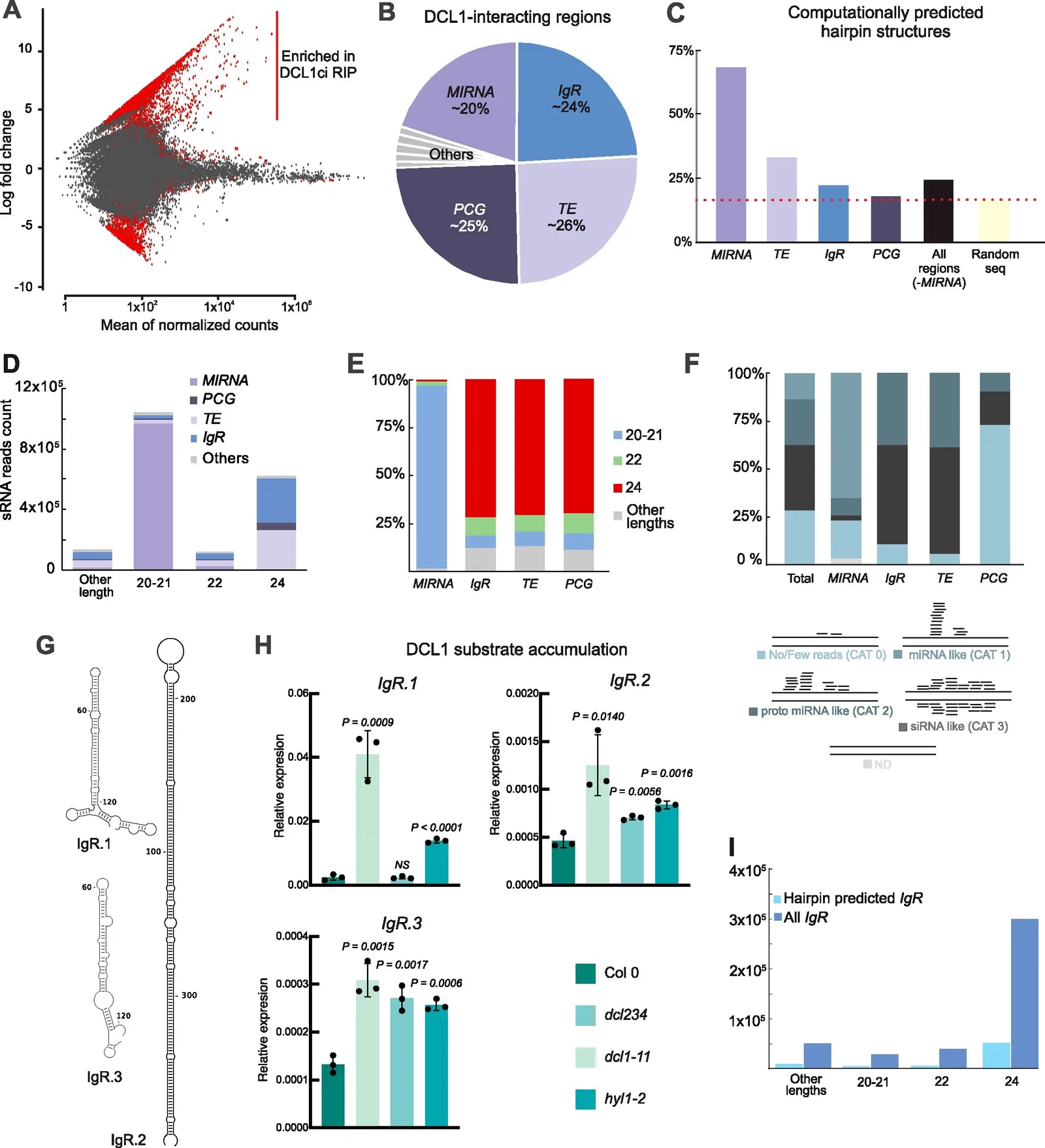
**(A)** MAplot representation of the differential analysis of reads count in *^DCL1^P-DCL1ci-HA/dcl1-7* versus *dcl1-7* over 100 bp windows covering the entire *A. thaliana* genome. Windows with an adjusted *P*-value <.05 are indicated in red. **(B)** Annotations of DCL1ci binding sites detected in panel (A) via DCL1ci-RIP. **(C)** Comparative proportions of DCL1ci-RIP-enriched loci identified and annotated in panels (A) and (B) containing predicted pre-miRNA-like stem-loop structures. The indicated percentage presents the proportion of stem-loop-containing DCL1ci binding sites against total annotated regions of each category. Stem-loops identified within random sequences provide an estimate of false positive frequency. **(D)** sRNA length from the DCL1ci-binding sites analyzed in panels (A)–(C). **(E)** sRNA length proportion within each annotation-types for regions enriched by DCL1ci-RIP in panels (A)–(C). **(F)** Categorization of the sRNA patterns associated with DCL1ci-binding sites. The top panel gives a graphical representation of the four categories (see the ‘Materials and methods’ section for details). The bottom panel shows the proportion of categories per annotation. **(G)** Examples of predicted hairpin structures from DCL1ci-RIP-enriched regions. **(H)** Quantitative RT-PCR (qRT-PCR) of transcript from hairpins represented in panel (G) in inflorescences of WT (Col-0), *dcl1-11, dcl234* (*dcl2-1/dcl3-1/dcl4-2)*, and *hyl1-2* plants. Actin2, RHIP1, and YLS8 were used as internal controls and for normalization. The graphs represent the average of three independent biological replicates involving each of ten individual inflorescences (at least two technical replicates). Error bars: standard deviation. Two-tailed, unpaired *t*-test (or Mann–Whitney test for the IgR1 results in *dcl234*) *P*-values are indicated. *NS*: nonsignificant difference. *n* = 3. Comparable results were obtained for a second independent experiment. **(I)** sRNA length distributions from all detected DCL1ci-interacting intergenic regions (dark blue) and those with a predicted stem-loop (light blue).

Annotation of the enriched regions (Fig. 3B) was done by comparing their genomic coordinates (intersectBed) to TAIR10 and miRbase V21 primary transcripts annotations (latest being enlarged by 100 bp up/downstream). When several annotations were overlapping a given region, the general annotation was given following a priority order (tRNA, rRNA, snoRNA, snRNA, miRNA_primary_transcript, inverted repeat, transposable element, pseudogene, ncRNA, protein coding genes).

#### sRNA-seq data processing

Small RNA sequencing data from WT and *dcl1-7* have been retrieved from SRA (accession number SRR764857 and SRR764859 respectively). Reads were filtered to 15- to 35-nt-long reads and matched against the Arabidopsis genome (TAIR10) using MUMmer v3.0 [48]. Only reads with a perfect match over their entire length were analysed further, allowing multiple mapping locations.

Number of 20–24-nt long reads nested into miRNA primary transcript annotation enlarged by 100 bp up/downstream were retrieve by genomic position comparison and used for miRNA family member comparison (Fig. 2E, G, and I) and for comparison between WT and *dcl1-7* (Fig. 2C) after normalization by the total number of mapped reads as well as their number of genomic positions (Fig. 2C).

For small RNA profile classification, sRNA sequences nested into the regions were define by genomic positions comparison and information relative to reads length, number of genomic positions, strand and sequence diversity (unique sequence versus number of reads) were retrieved. This information was used for classification as follows:

No-Few reads: <5 reads per 100 bp
siRNA-like: at least 5 reads per 100 bp and <4 times more reads on one strand in comparison to the other
proto-miRNA: at least 5 reads per 100 bp, 4 times more reads on one strand than the other, the most abundant sequence corresponds to maximum 40% of the total number of reads from the regions
miRNA-like: at least 5 reads per 100 bp, 10 times more reads on one strand than the other, the most abundant sequence corresponds to >40% of the total number of reads from the regions, at least 1 sequence with only one genomic position.

#### Pre-miRNA-like secondary structures prediction

The analysis for identifying putative new miRNA precursors was conducted using an in-house script developed with the FORGI library (version 2.0.0). This script was used to scan the DCL1ci binding sites detected to identify those that could fold into hairpin structures similar to plant miRNA precursors. Based on previously described plant miRNA precursors, we set criteria for a stem of at least 25 base pairs, allowing a maximum of 10 mismatches within this region. Furthermore, the random sequences employed in the analysis were genomic regions that maintain the same distribution of lengths as the original DCL1ci binding sites under study.

#### AGO1-IP sRNA sequencing data and target prediction

To identify AGO1 loaded sRNA reads nested in DCL1ci enriched regions we used our previously published AGO1-IP sRNA sequencing data (GSM2680245 and GSM2680246). After curation based on their abundance, number of genomic positions and 5′ nucleotide termini, the remaining sequences were used for target prediction using psRNAtarget (https://www.zhaolab.org/psRNATarget/) on *A. thaliana* TAIR10 Transcript Library (removed miRNA gene) with default parameters. Several predicted targets were then selected for further validation.

#### *IR71* secondary structure prediction

Genomic sequence corresponding to the regions Chr3: 1962 100–1972 099 was extracted from TAIR10 refence sequence reverse-complemented. A local version of RNAfold v2.4.18 was used for secondary structure prediction and visualization (parameters: -d2 –noLP -T 24) PMID [49]. Visualization of local secondary structure were generated using the Vienna RNA Websuite with default parameters [50].

#### Bisulfite sequencing data analysis

Processed data (.wig files) were retrieved from Gene Expression Omnibus for the mutants of interest (accessions GSE39901 and GSE70912). For each cytosine sequence context, methylation levels of cytosine corresponding to our regions of interest were retrieved using intersectBed function from bedtools, average methylation level was calculated for each region and represented as boxplot using R (Supplementary Fig. S4F).

#### Sequences comparison and multiple alignments.

Multiple alignments of miRNA family stem-loop sequences are based on miRbase V21 primary transcript sequences and have been done and visualized with Clustal Omega (Default parameters) and Jalview [51].

#### *DCL1* 5′ UTR conservation and secondary structure

https://phytozome-next.jgi.doe.gov/ v13 was queried for DICER in all 29 available Brassicaceae species. When several genome versions were available, only the most recent one was used and when the queried reported more than one Dicer one homologue were listed (i.e. *Indigofera tinctoria* v1.1 Isati.0731s0010 and Isati.0077s0036) only one was considered. The genomic sequences from the reported hit were retrieved with 500-nt upstream (*Spondias pinnata* v1.1 was discarded as no upstream sequence was available (beginning of the scaffold). Sequences were then aligned with Clustal Omega and visualized with Jalview. Principal Component Analysis (PCA) indicates that *Euclidium syriacum, Dendrocalamus strictus*, and *Cattleya violacea* appears at outliers and were therefore removed from further analysis. For the remaining 25 species, Sequences upstream of the Arabidopsis ATG start codon were extracted and subsequently realigned with R-coffee for precise identification of the similarity with the UTR regions from *AT1G01040.1*.

Local secondary structures were predicted with scanFold [52] using a window of 80-nt and a temperature of 24°C. Graphical representation were generated with RNAfold [53]. Sequences from the multiple alignment corresponding to the identified stem-loop were extracted and analysed for sequence and structure conservation with LocARNA (PMID 22450757) from Freiburg RNA tools [54]. Global folding of the UTR regions from *AT1G01040.1* was done with RNAfold (temperature 24°C) and comparison of the 25 Brassicaceae *DCL1* 5′UTR sequence and secondary structure was done using CARNA (PMID 22689637) and locARNA.

### Protein blot analysis

Protein concentrations were determined by a modified Lowry procedure using the DC^TM^ Protein Assay Kit (Bio-Rad). Proteins were resolved by sodium dodecyl sulphate–polyacrylamide gel electrophoresis and electro-transferred to Immobilon-P polyvinylidene difluoride (PVDF) membrane (Millipore). Following a 1 h blocking step in 1× Tris-buffered saline (TBS) + 0.1% Tween-20 supplemented with 5% nonfat dry milk, antibody incubations were carried out overnight at 4°C with constant shaking (Supplementary Table S8). Membranes were then washed three times in TBS + 0.1% Tween-20, subsequently incubated for 1h in horseradish peroxidase-conjugated goat anti-rabbit secondary antibody, then rinsed three times before detection with the ECL Western Blotting Detection Kit (GE Healthcare Life Science) under a ChemiDoc^TM^ Touch imaging system (Bio-Rad).

### Real-time qRT-PCR and sqRT-PCR analyses

Total RNA was extracted from frozen tissue and ground with pestle and mortar in TRIzol (Invitrogen) following the manufacturer’s instructions. Total RNA was treated with DNaseI (Thermo Fisher Scientific) for 30 min at 37°C before reverse transcription with the Maxima First Strand cDNA Synthesis Kit (Thermo Fisher Scientific). sqPCRs were performed using the DreamTaq DNA polymerase (Thermo Fiser Scientific) and the indicated primers (Supplementary Table S8). Real-time qPCRs were performed on a LightCycler480 II (Roche) with the KAPA SYBR^®^ FAST for LightCycler^®^480 qPCR Kit (KAPA Biosystems) and the indicated primers (Supplementary Table S8), following the PCR program recommended with the KAPA SYBR FAST qPCR mix. In addition, a melting curve was generated at the end of the amplification. Threshold cycle (C_p_) values were determined by calculating the second derivative maximum of the amplification curves using the LightCycler 480 software. Relative expression levels were calculated by subtracting the average C_p_ values for the genes of interest to housekeeping genes as listed in the figure legends (to give ∆C_p_) and then calculating ‘2^∆Cp^’. Oligos used for qPCR (Supplementary Table S8).

### *Agrobacterium tumefaciens*-mediated protein transient expression

For protein transient expression assays in *rdr6i N. benthamiana*, binary vectors were introduced into *Agrobacterium tumefaciens* GV3101 through electroporation. The transformed bacteria were grown at 28°C in Luria–Bertani medium supplemented with the appropriate antibiotics. The bacterial cultures were collected and normalized to an OD600 of 0.15–0.5 using a solution containing 10 mM MgCl_2_, 10 mM 2-(N-morpholino)ethanesulfonic acid (MES) buffer, pH 5.6, and 150 μM acetosyringone. These suspensions were then infiltrated into the abaxial side of 2-week-old *N. benthamiana* leaves. When simultaneous expression of two proteins was required, cultures containing the corresponding vectors were mixed prior to infiltration.

## Results and discussion

### Catalytically inactive DCL1 interacts with the microprocessor component HYL1 and stabilizes co-immunoprecipitated pri-miRNAs

To temporarily stabilize interactions between DCL1 and its substrates, we introduced point mutations on each catalytic site (E1508Q and E1697Q) of the RNase III domain to generate *DCL1ci* (Fig. 1A, and Supplementary Fig. S1A and B). Genomic sequences of wild-type *DCL1* (*DCL1wt*) and *DCL1ci* were cloned under the *DCL1* promoter and C-terminally fused translationally to the Human influenza HA epitope. Both were transformed into hypomorphic *dcl1-7* mutant Arabidopsis to generate the *^DCL1^P-DCL1wt-HA/dcl1-7* and *^DCL1^P-DCL1ci-HA/dcl1-7* genotypes, respectively. While the strong developmental defects caused by the *dcl1-7* mutation were reversed in *^DCL1^P-DCL1wt-HA/dcl1-7* plants, they persisted in *^DCL1^P-DCL1ci-HA/dcl1-7* plants across all tissues and developmental stages. Accordingly, near-wildtype miRNA levels were restored in the former, whereas they remained at, or below detection in the latter, as in noncomplemented *dcl1-7* plants (Fig. 1B and C, and Supplementary Fig. S1C). By contrast, the levels of the DCL4-dependent miR822 and DCL-independent small nuclear RNA U6 were similar in *^DCL1^P-DCL1wt-HA/dcl1-7, ^DCL1^P-DCL1ci-HA/dcl1-7, dcl1-7* and WT plants (Supplementary Fig. S1C). In colocalization and bimolecular fluorescence complementation (BiFC) assays, DCL1ci colocalized and interacted with the microprocessor protein HYL1 similarly to DCL1wt (Fig. 1D and Supplementary Fig. S1D and E), suggesting that DCL1ci maintains the ability to recognize known interacting partners and can indeed be used to stabilize pri-miRNAs and other possible substrates.

This idea was validated in RIP experiments conducted in inflorescences of *^DCL1^P-DCL1wt-HA/dcl1-7* and *^DCL1^P-DCL1ci-HA/dcl1-7* Arabidopsis (Fig. 1A). Semi-quantitative reverse transcription sqRT-PCR analyses revealed that the latter indeed granted a strong enrichment of several DCL1-dependent pri-miRNAs tested (Fig. 1E). RIP-Seq was conducted in biological triplicates using pooled inflorescences from*^DCL1^P-DCL1ci-HA/dcl1-7* and *dcl1-7* plants. As a proof-of-principle that RIP-Seq conducted with DCL1ci enables better stabilization of DCL1 substrates than that theoretically granted by direct RNA-Seq in the *dcl1-7* background, we first examined all known *MIRNA* loci. Following alignment onto the Arabidopsis genome (TAIR10), RNA reads corresponding to all annotated pri-miRNAs (miRbase v21) were counted and used for differential analysis (see the ‘Materials and methods’ section); annotated tRNA and rRNA genes were included as negative controls. Out of 325 annotated pri-miRNA transcripts, 100 (30.7%) were detected as significantly enriched in DCL1ci-RIP compared to total RNA from *dcl1-7* inflorescences (P.adj < 0.05; Fig. 1F and Supplementary Table S1). Only seven pri-miRNAs with an average normalized reads count > 100 were not detected as enriched in DCL1ci-RIP. Of these, at least two (pri-miR163 and -miR824) are likely false negatives caused by variability between replicates. Another two (pri-miR4228 and -miR5642b) are unlikely *bona fide* pri-miRNAs, as strongly suggested by curation on miRbase. By contrast, tRNAs and rRNAs were not significantly enriched in DCL1ci-RIP (Fig. 1F, Supplementary Fig. S1D, and Supplementary Table S1). A refined analysis considering evolutionary conservation revealed that the enrichment was more pronounced on ancient- (56/93) compared to young- (44/188) pri-miRNAs. This is likely explained by the DCL1-only processing reliance of the former compared to the more promiscuous processing (including by DCL4) of the latter (Fig. 1G and Supplementary Table S2). At least one member of 96% of all evolutionarily conserved *MIRNA* families was detected, indicating the high sensitivity of the DCL1ci approach despite it being only applied to inflorescences here (Fig. 1H and Supplementary Table S2). Publicly available sRNA-Seq data from WT Arabidopsis inflorescences [55] confirmed that the pri-miRNAs identified via DCL1ci spawn nearly exclusively 21-nt-long sRNAs (Fig. 1I and Supplementary Table S2). We conclude that DCL1ci displays molecular attributes compatible with the transcriptome-wide exploration of DCL1’s substrates, as opposed to products, using RIP-Seq.

### DCL1 likely scans pri-miRNAs to identify and subsequently process pre-miRNAs via several possible mechanisms

The global distribution profiles of RIP-Seq signals on *MIRNA* loci were qualitatively highly reproducible in the three biological replicates (Fig. 2A). Analyzing individual *MIRNA* loci revealed consistent DCL1ci enrichments not only at the expected pre-miRNA stem-loops but also over several hundred pri-miRNA-encompassing nucleotides located upstream and/or downstream thereof (Fig. 2A). Log-scale visualization indicates that these enrichments were generally less pronounced, however, than at pre-miRNA stem-loops. Given the use of formaldehyde crosslinking in the procedure, indirect and spurious DCL1ci-RNA contacts cannot be formally excluded. We consider them unlikely, however, because the observed RIP-seq profiles were not only discrete but also highly reproducible across experimental replicates (Fig. 2A and Supplementary Fig. S2C) unlike what would be expected from indirect, random interactions. Since DCL1ci RIP typically captures up to ∼200-nt of RNA surrounding the DCL1ci interaction sites (see the ‘Materials and methods’ section), these observations suggest that, while considered primarily as a dsRNA-binding protein, DCL1 may interact with single-stranded regions of pri-miRNAs to perhaps enable rapid scanning of pre-miRNA stem-loops. Their recognition might then activate DCL1’s RNase III activity to initiate miRNA biogenesis. How DCL1 might be recruited onto pri-miRNA transcripts (via binding to their 5′m7G cap and/or to pri-miRNA-intrinsic sequences, or else) is an interesting question for future investigations.

Our analysis also revealed that reads from DCL1ci-RIP repeatedly covered both the guide and passenger miRNA (miRNA*) strands (Fig. 2B and Supplementary Fig. S2A), with the latter being usually at/below detection of conventional sRNA-Seq analyses. For some pre-miRNAs, the higher DCL1ci interaction peak was on guide miRNA strands, as for miR162a, miR165a, miR172bcd, miR396b and miR408. For others, however, it corresponded to the miRNA* strands, as for miR166a, miR168b, miR170, miR319a, and miR390. In a third scenario, the higher DCL1ci interaction peak was in the vicinity of either the guide or passenger strands, as for miR159a, miR160a, miR164b, miR167a, and miR399a (Fig. 2B and Supplementary Fig. S2A). These differences observed on DCL1’s main pre-miRNA-interaction zones could be explained by the heterogeneity of plant miRNA precursors in terms of their sizes, structures, or the 5′/3′-strand location of miRNAs [33]. They may also reflect the recognized diversity of plant miRNA processing mechanisms including via simple versus sequential cleavages in the base-to-loop versus loop-to-base orientation, for instance [33]. Consistent with this idea, analysis of DCL1ci-RIP peak positions relative to annotated precursors revealed a significant association between DCL1’s binding geometry and miRNA processing mechanism (Fisher’s Exact Test, *P* = 0.0146). For precursors processed in a loop-to-base manner (including both short and sequential types), the most enriched DCL1ci-RIP peaks were predominantly located within the pre-miRNA (94.4%). By contrast, the most enriched DCL1ci peaks showed a much higher tendency to occur in flanking regions (37.7%) for base-to-loop precursors (including both short and sequential types), supporting distinct positional modes of DCL1 engagement (Supplementary Fig. S2B). These results suggest that, in base-to-loop precursors, DCL1 may initially associate and pause in the flanking regions upon encountering the stem-loop, likely because it cannot perform the first cleavage at the base. In contrast, for loop-to-base precursors, DCL1 binding is predominantly confined within the pre-miRNA region, probably reflecting its inability to initiate cleavage in the loop region, leading to stable association within the hairpin. Altogether, these findings support a model in which DCL1 scans pri-miRNAs to identify and subsequently process pre-miRNA hairpins through distinct base-to-loop and loop-to-base mechanisms.

### DCL1ci qualitatively and quantitatively discriminates paralogous pri-miRNAs

Duplication drives plant *MIRNA* gene evolution leading to expansion of multicopy miRNA familie [56, 57]. In Arabidopsis more than one third of *MIRNA* genes are from segmental duplication, and 50% of inflorescence *MIRNAs* identified in our survey indeed map to multiple loci with copy numbers as high as 14 (Fig. 2C). Within each *MIRNA* family, paralogous pre-miRNAs typically yield identical or near-identical mature miRNA sequences, despite substantial sequence divergence along the surrounding pre-miRNA regions (Fig. 2D and Supplementary Fig. S2C). Consequently, in conventional sRNA-Seq analyses, the accumulation of mature miRNAs in a given tissue cannot be unambiguously attributed to specific paralogous pri-miRNAs expressed therein. Instead, multimapping causes identical sRNA reads to be equally and erroneously assigned to all paralogous pre-miRNAs that produce indistinguishable mature miRNAs. Conventional sRNA-Seq also falls short of quantifying pri-miRNA levels due to their short half-life reflecting the rapidity of the miRNA processing steps [58, 59].

Use of DCL1ci RIP simultaneously identified all 22 evolutionarily conserved *MIRNA* paralogous families know to contribute mature miRNA production in inflorescences (Fig. 2C and Supplementary Table S2). Global analysis further revealed instances (such as for *MIR156, MIR157, MIR160, MIR165, MIR166, MIR167, MIR168*, and *MIR390*, among others) where DCL1ci interacted with one or only a subset of paralogous pri-miRNAs (Fig. 2C and Supplementary Table S2). For example, in the two-members *MIR162* family, both loci a and b can in principle contribute the same mature miRNA 5′UCGAUAAACUGCAUCCAG3’. However, of the two, *MIR162b-*derived pri-miRNA mostly interacted with DCL1 in inflorescences (Fig. 2C–F). A similar observation was made with the seven *MIR166* family members encoding the same mature miRNA 5′UCGGACCAGGCUUCAUUCCCC3’. Indeed, only *MIR166a-* and *MIR166b*-derived pri-miRNAs interacted with DCL1ci in this case (Fig. 2C, D, G, and H). Figure 2C therefore provides the first exhaustive and unbiased repertoire of pri-miRNAs quantitatively and qualitatively available for mature miRNA production in a given plant tissue. While pools of inflorescences were used here to establish a proof-of-concept, the method could be applied to other plant tissues, developmental stages and/or under various stress conditions. Moreover, expressing DCL1ci under cell-type-specific promoters would allow quantitative and qualitative pri-miRNA discrimination at cellular resolution. Nonetheless, while this approach provides an unbiased detection of pri-miRNAs effectively recognized by DCL1, we cannot exclude the possibility that certain precursors are selectively recognized and degraded by other factors prior to DCL1 engagement, nor that all detected interactions reflect equally efficient processing.

Alternative methods have been developed for pri-miRNA mapping in specific tissues, including, primarily, laser-capture microdissection (LCM) coupled to pri-miRNA quantification by qRT-PCR [60]. Yet, arguably, LCM primarily captures the most stable, i.e. least processed pri-miRNAs, thereby possibly missing the most biologically relevant molecules in investigated tissues. The less technically challenging and less tissue-invasive DCL1ci-RIP approach overcomes this caveat because it captures pri-miRNAs before processing *in planta*. Combined with cell-specific mature miRNA quantification systems already in place in Arabidopsis [61], the method may ultimately enable accurate parallel quantification of pri-miRNA versus mature miRNA levels cell-specifically. This could help uncover post-transcriptional regulatory events suspected to occur during the successive steps of miRNA biogenesis [62, 63], perhaps in a *MIRNA*-specific manner, and thereby reveal the extent to which pri-miRNA levels may be used to extrapolate mature miRNA accumulation. Regardless, the availability of the *^DCL1^P-DCL1ci-HA/dcl1-7* line now makes it possible to uncover specific biological functions exerted by certain *MIRNA* paralogs in certain cell types or under some, unlike other, conditions. In principle, it could also help investigate mature miRNA mobility between tissues or cell types although pri-miRNA movement has been documented from cells/tissues naturally exhibiting reduced pri-miRNA processing [61, 64].

### DCL1ci accurately detects low-expressed, evolutionarily young *MIRNA*- and “transitioning” proto-*MIRNA*- loci

miRNAs conserved across species are generally abundantly expressed. Most species-specific miRNAs, by contrast, accumulate at low levels [65] and hence, little is known about their expression, biogenesis, and DCL-dependency. DCL1ci RIP detected 44 (i.e. ∼21%) of all described evolutionarily young *MIRNA loci* (Fig. 2I and Supplementary Table S2). Of these 44, only 20 showed a significant number (>50) of mature miRNA reads in Deep-seq data. Thus, DCL1ci-RIP identifies sRNA-producing loci with a higher sensitivity than classical sRNA sequencing, independently of the sRNA stabilization potentially achieved through AGO loading [66]. Analyzing individual young *MIRNA* loci also revealed DCL1 interaction with single-stranded pri-miRNA regions surrounding the predicted pre-miRNA stem-loops, supporting DCL1 scanning (Supplementary Fig. S2D) as observed with conserved *MIRNA* loci (Fig. 2B and Supplementary Fig. S2A). Variations in the main regions of interaction within pre-miRNAs were also observed (Supplementary Fig. S2E), as in Fig. 2B and Supplementary Fig. S2.

While most of the young *MIRNA* loci detected produce mainly 21-nt sRNAs in a DCL1- or DCL4-dependent manner as described previously (Fig. 2J and Supplementary Table S2) [31], others such as *MIR776* and *MIR5356* appear to produce mainly 24-nt sRNAs in a DCL1-independent (Supplementary Table S2) and likely DCL3-dependent manner. One hypothesis pertaining to their origin contends that *MIRNA* loci are initially inverted-duplications producing perfectly paired stem-loop RNA processed chiefly by siRNA-generating DCLs. Over time, such proto-*MIRNAs* would accumulate mutations yielding an increasing number of mismatches along the precursor’s stem. The ensuing imperfect fold-back precursors would then progressively undergo preferential processing by DCL1 [27, 67, 68]. Our results reveal that DCL1ci can be used to accurately detect low-expressed evolutionarily young *MIRNAs* loci as well as such “transitioning” proto-*MIRNA*s, as indeed shown later in this work.

### DCL1ci is enriched at distinct regions of the endo-*IR* representative *IR71*, and might contribute distinctively to sRNA production therefrom

Transcribed endogenous inverted-repeats (*IRs*) resemble evolutionarily young *MIRNA* and proto-*MIRNA* loci, yet, likely due to their size/structure, are processed by DCL2, DCL3, and/or DCL4 albeit with DCL1’s assistance via unclear mechanisms [5, 11, 69]. *IR71* is a well-characterized, long (∼3-kb dsRNA) *IR* hierarchically processed into siRNAs by DCL2 and DCL3, and secondarily by DCL4. Accordingly, *IR71* spawns mainly 22- and 24-nt sRNAs alongside a minor population of 21-nt sRNAs. Among the loci detected via DCL1ci RIP, four regions (*IR71.1*→*4*) overlapped *IR71* and showed a weak yet significant enrichment (Supplementary Fig. S3A and Supplementary Table S3). This indicates DCL1 interaction with *IR71*-derived transcripts and suggests DCL1’s contribution to the processing of *IR71* dsRNA into sRNAs. Indeed, northern analysis revealed that all three major *IR71*-sRNA length populations (21-nt, 22-nt, and 24-nt) decrease in abundance in *dcl1-11* versus WT backgrounds (Fig. 2K). Mining deep-seq data confirmed this observation and the DCL2-, DCL3- and DCL4-dependency of sRNAs (Supplementary Fig. S3B). The hypomorphic *dcl1-11* allele facilitated parallel analyses without overt developmental anomalies. sRNA levels were also reduced in *hen1–6* mutant tissues, agreeing with the 3′ ends of both plant miRNAs and siRNAs being protected by HEN1-catalysed 2′-O-methylation (Fig. 2K).

A closer look at the four enriched regions revealed that *IR71.1* and *IR71.2* share sequence similarity and indeed strongly base-pair with each other in the context of the overall *IR71* dsRNA structure (Supplementary Fig. S3A). The two other detected DCL1ci-interacting regions, *IR71.3* and *IR71.4*, are dissimilar in sequence. While both are embedded on the RNA strand yielding the overall *IR71* secondary structure (Supplementary Fig. S3A), they generate local hairpin structures with high-confidence scores (Supplementary Fig. S3C), unlike *IR71.1* and *IR71.2*. Strikingly, while the sRNA signal was below detection in *dcl234* for *IR71.1* and *IR71.2*, a respectively weak and strong 21-nt signal was detected for *IR71.3* and *IR71.4* (Fig. 2K), which suggests that DCL1 activity is either lacking or significantly reduced on *IR71.1* and *IR71.2*. What this activity might be was hinted at by the observation that the sRNA signal remained unchanged in the *hyl1* mutant background for *IR71.1* and *IR71.2*, whereas that from the two remaining regions was partially (*IR71.3*) or fully *(IR71.4*) HYL1-dependent (Fig. 2K). This specific HYL1-dependency could suggest that the local hairpin structures of *IR71.3* and *IR71.4* are akin to pre-miRNAs and perhaps recruit DCL1 for pri→-pre-miRNA processing-like reactions. This absent initial step on *IR71.1* and *IR71.2* might predispose *IR71.3* and *IR71.4* to substantially stronger interactions with DCL1, possibly explaining the persistence of a 21-nt signal derived thereof *in dcl234*, unlike from *IR71.1* and *IR71.2* (Fig. 2K). We showed previously that heat shock (HS, 37°C) strongly induces *IR71* transcription and increases accumulation of *IR71*-derived siRNAs with systemic regulatory potential [70]. It will be therefore interesting to test if HS also modifies the repartition of DCL1ci onto *IR71*, which could potentially reveal HS-induced dsRNA melting-re-annealing events. These may modify/create local hairpin structures akin to *IR71.3 -4* that possibly impact the *IR71*-siRNA landscape and hence, *IR71*’s systemic regulatory functions. DCL1ci-based mapping under HS could reveal if *IR71-* and perhaps other *IR*-derived transcripts are conceptually similar to RNA thermo-sensors [71, 72].

### A broad range of DCL1-interacting loci generate sRNAs diverging in length and accumulation patterns form those of bona-fide miRNA precursors

Having focused on *MIRNA* and *IR* loci, we then asked if RNA transcribed from other genomic regions might be enriched in DCL1ci RIPs. We applied genome-wide enrichment analyses on 100 bp windows spanning the entire genome (see the ‘Materials and methods’ section). This identified 5035 windows with significant RNA reads enrichments in the three *^DCL1^P-DCL1ci-HA/dcl1-7* RIP biological replicates compared with the three *dcl1-7* RIP controls (Fig. 3A). By selecting all normalized reads count >50 in DCL1ci RIP and merging the corresponding nearby windows, we identified 470 DCL1ci-interacting genomic locations with a ∼240 bp average length. As expected, *MIRNA* genes represented a significant fraction (20.2%) of these loci. yet even higher fractions mapped to protein-coding genes (*PCGs;* 24.7%), transposable elements (*TEs*) (25.5%) and intergenic regions (*IgRs*; 23.8%). Finally, an “other loci” category, including *IRs*, represented 5.8% of detected regions (see the ‘Materials and methods’ section; Fig. 3B and Supplementary Table S3).

To further characterize these DCL1ci-interacting genomic loci, we computationally scanned the corresponding DCL1ci binding sites for stem-loop structures akin to those of plant pre-miRNAs, using conserved *MIRNAs* to estimate false negatives (see the ‘Materials and methods’ section). Out of the 470 DCL1ci-binding sites, 157 (33%) could form stable pre-miRNA-like secondary structures (Supplementary Table S4). A total of 63 out of 95 (66%) evolutionary conserved known *MIRNA* loci identified with DCL1ci also contained a secondary structure (Fig. 3C). The stringency of our prediction of stem-loop structures, as well as the size and structural heterogeneity of plant compared to animal pre-miRNAs [32] likely contributed to the 34% false negative rate observed in this case. Nonetheless, these two features unlikely explained the relatively high percentage of DCL1ci-enriched regions in which no stable secondary structures could be identified according to our criteria. Indeed, after excluding known *MIRNA* loci, only 94 out of 375 (25%) DCL1ci-enriched regions formed stable hairpin structures. More specifically, this feature was merely detected on 33% (40/120), 19% (22/116), and 23% (16/112) of *TEs, PCGs*, and *IgRs*, respectively (Fig. 3C and Supplementary Table S4). A total of 63 out of 382 (16%) random genomic sequences, used as negative controls, formed secondary structures according to our criteria (Fig. 3C). Collectively, these results suggest that DCL1 binding to RNA is much less stringently conditioned by secondary structures than originally anticipated. This is consistent with the proposed scanning of DCL1 on ssRNA regions of pri-miRNAs.

Exploiting publicly available deep-seq data from WT Col-0 tissues, we analyzed the abundance, length and accumulation patterns of sRNAs generated from the 470 DCL1ci-interacting genomic loci. As expected, high levels of 20–21-nt sRNAs were produced from annotated *MIRNA* loci (Fig. 3D and Supplementary Table S5). Surprisingly, however, abundant 24-nt sRNA reads mapped to *TEs, PCGs*, and *IgRs* (Fig. 3E and Supplementary Fig. S3D). sRNA accumulation patterns were classified based on the most abundant sequences and their strand bias, if applicable, leading to four categories (CATs). CAT1 encompasses *MIRNA*-like loci defined by one dominating discrete sequence with a strong strand bias; CAT2 encompasses *proto-MIRNA* loci displaying a strong strand bias but no dominating sequence; CAT3 encompasses loci associated with siRNA-like populations lacking overt strand biases. CAT0, finally, groups regions with <5 reads per 100 bp (Fig. 3F, Supplementary Fig. S3E, and Supplementary Table S5; see the ‘Materials and methods’ section). As expected, conserved *MIRNA* loci mostly populated CAT1, and young *MIRNAs* were enriched on CAT2. (Fig. 3F and Supplementary Fig. S3E). By contrast, sRNA accumulation patterns in *TEs* and *IgRs* fell into CAT3 (Fig. 3F). A total of 30% of *PCGs* showed a high proportion of 24-nt reads like *TEs* and *IgRs* (CAT3; Fig. 3E and F) but, curiously, the remaining 70% had no or few sRNA reads (CAT0; Fig. 3F). Altogether, these results suggest that DCL1 interacts with a broad spectrum of genomic locations generating sRNAs whose length and accumulation patterns diverge from those described for *bona-fide* miRNA precursors. These hitherto unrecognized and atypical features could explain why *TEs, PCGs*, or *IgRs* were never connected to DCL1 using canonical approaches such as sRNA deep seq analysis or secondary structures’ predictions.

### *IgRs* contain hitherto unknown proto-MIRNAs

Given that *MIRNA* genes are often located within *IgRs*, we investigated further if the 24% fraction detected above might encode previously nonannotated *MIRNAs*, as anticipated from the previous section. Analysis of pre-miRNA-like hairpins predicted in DCL1ci-RIP-associated *IgRs* indeed revealed a heterogeneous collection of stem-loops of variable sizes (Fig. 3G, Supplementary Fig. S3F, and Supplementary Table S4). Confirming that they are *bona fide* DCL1 substrates, these transcripts over-accumulated in the *dcl1* and *hyl1* miRNA-defective mutants, as assessed by RT-qPCR and sqRT-PCR analyses (Fig. 3H and Supplementary Fig. S3G). Interestingly, the transcripts also over-accumulated in the siRNA-defective *dcl234* triple-mutant, albeit not to the level seen in the *dcl1* single mutant (Fig. 3H). This suggested their processing prominently by DCL1 with assistance of one or several siRNA-producing DCLs. Supporting this notion, analysis of sRNA seq data revealed that most of these intergenic regions spawn predominantly 24-nt sRNAs presumably produced by DCL3 (Fig. 3I). Twenty-one nucleotide sRNA reads mapping these regions were also detected in sRNA libraries prepared from AGO1-IPs (Supplementary Table S6). Nonetheless, most of these newly identified proto-*MIRNAs* (and putative targets) are likely too lowly expressed to be detected without direct Dicer binding, granted here by the DCL1ci allele. Further analysis will be required to elucidate the biological role(s), if any, of these evolutionary recent loci. Altogether these results confirm that DCL1ci can be used to accurately detect novel low-expressed *MIRNAs* loci as well as “transitioning” proto-*MIRNA*s loci.

### DCL1 interacts with *TE*-derived RNA and promotes siRNA accumulation therefrom in a manner antagonizing RdDM

**Figure 4.**
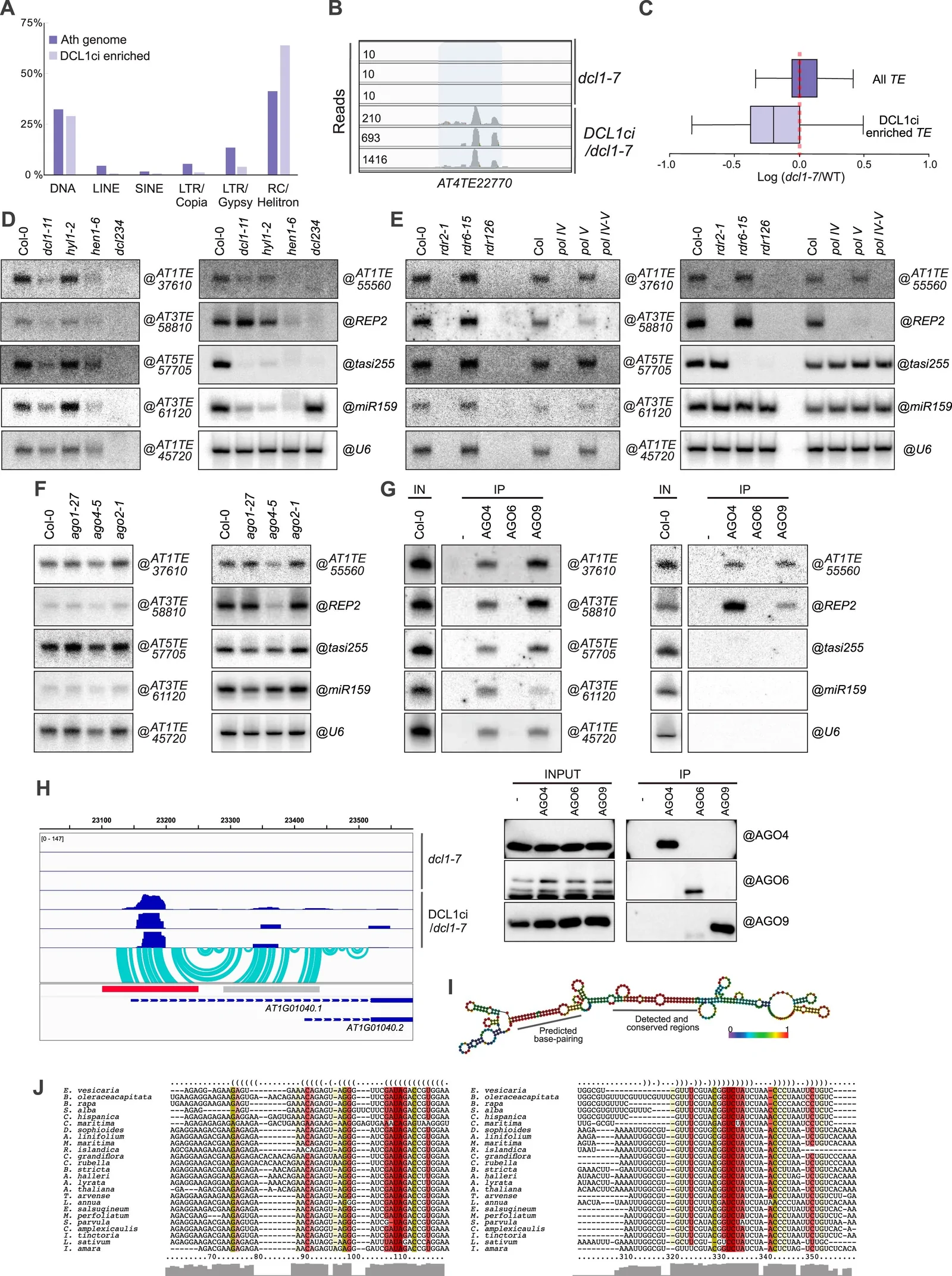
**(A)** Analysis of *TEs* super-families annotated in the *A. thaliana* genome and those identified via DCL1ci-RIP. **(B)** IGV visualization of reads coverage from three replicates of *^DCL1^P-DCL1ci-HA/dcl1-7* versus *dcl1-7* control plants over an Helitron (*AT4TE22770; ATREP*15) detected by DCL1ci-RIP. **(C)** 24-nt sRNA reads accumulations in wild-type versus *dcl1-7* mutant libraries from *TE* regions detected via DCL1ci-RIP versus all *TE* annotated in the *A. thaliana* genome. **(D)** Northern blot analysis of 24-nt sRNAs arising from six *TE* regions detected in the DCL1ci-RIP in WT (Col-0), *dcl1-11, hyl1-2, hen1-6*, and *dcl234* (*dcl2-1/dcl3-1/dcl4-2*) Arabidopsis inflorescences. Rep2, tasi255, miR159, and U6 are shown as controls. The membrane was stripped and re-probed multiple times. **(E)** Northern blot analysis of 24-nt sRNAs arising from the six *TE* regions in WT, *rdr2-1, rdr6-15, rdr126* (*rdr1-1/rdr2-1/rdr6-15), pol IV (nrpd1a), pol V (nrpd1b)*, and *pol IV(nrpd1a)-polV(nrpd1b)* Arabidopsis inflorescences. Same controls as in panel (D). **(F)** Northern blot analysis of 24-nt sRNAs arising from the six *TE* regions on WT, *ago1-27, ago2-1*, and *ago4-5* Arabidopsis inflorescences. Same controls as in panel (D). **(G)** Northern blot analysis of 24-nt sRNAs arising from the six *TE* [and same controls as in panel (D)] in IP fractions of AGO4, AGO6, and AGO9 (upper panels). Western blot analysis validating AGO4, AGO6, and AGO9 IPs (lower panels). Same controls as in panel (D). **(H)** IGV visualization of reads coverage from RNA co-IPed in *^DCL1^P-DCL1ci-HA/dcl1-7* or *dcl1-7* over the *DCL1* mRNA’s 5′UTR. The red rectangle indicates the regions detected as significantly enriched in the DCL1ci-RIP; and the gray rectangle indicates the regions detected significantly enriched in the DCL1ci-RIP which did not pass our minimal-reads cutoff. The cyan arcs indicate the predicted base pairing of *DCL1*’s 5′ UTR sequences between both windows. **(I)** Secondary structure predicted with RNAfold using the *DCL1* 5′ UTR sequence representing predicted base pairing in Fig. 4H. **(J)** Conservation of the stem from the *DCL1* 5′ UTR structure across inspected Brassicaceae.

A total of 120 (25%) of the loci interacting with DCL1ci overlap *TEs* (Fig. 3B) with corresponding sRNA accumulation patterns consistent with DCL3-dependent 24-nt siRNA populations (Fig. 3E). Eukaryotic *TEs* are classified based on their transposition mechanisms [73] and specific sRNA silencing pathways are known to target specific *TE*-classes in plants [74, 75]. Exploring the DCL1ci-enriched *TE* superfamilies against full genome annotations revealed an overrepresentation of helitrons (Fig. 4A and B). While some helitrons contain 3′ short stem-loops [76], DCL1ci was not specifically enriched over these regions. We compared the sRNA reads accumulation in WT and *dcl1-7* for all *TE* annotations and more specifically for the *TEs* detected in DCL1ci-enriched regions with overrepresented helitrons. Globally, 24-nt sRNA abundance from all annotated *TEs* did not overtly change in *dcl1-7* versus WT backgrounds. Twenty-four-nucleotide sRNAs produced from DCL1ci-renriched *TE*s, by contrast, had reduced levels in *dcl1-7* (Fig. 4C and Supplementary Table S7). This was confirmed by northern analysis performed on several candidates (Fig. 4D) also validating no decrease for the canonical DCL3-dependent *REP2*-derived 24-nt siRNAs in *dcl1-7* (Fig. 4D).

As expected, the sRNA signal was reduced or below detection in *hen1-6* and *dcl234* (Fig. 4) for both DCL1ci-renriched *TEs* and *REP2*. More surprisingly, the 24-nt sRNA levels from the *TE* candidates were unchanged in *hyl1-2*, unlike accumulation of sRNAs known to be directly (e.g. miR159) or indirectly (e.g. tasi255) DCL1-dependently processed (Fig. 4D). This lack of *hyl1-2* effect makes it also unlikely that sRNA production from the identified DCL1ci-interacting *TEs* resembles that of ‘epigenetically activated’ siRNAs (easiRNAs), which requires targeting of certain *TE* transcripts by DCL1- and HYL1-dependent miRNAs [75]. Furthermore, we did not observe major changes in 24-nt sRNA levels in either *ago1-27* or *ago2-1* mutants known to compromise miRNA steady-state levels and activity, which are required for easiRNAs biogenesis (Fig. 4F). Finally, while 24-nt sRNA levels were strongly reduced in *rdr2-1* and *pol IV* (*nrpd1a*) mutants, no change was observed in the *pol V* (*nrpd1b*) mutant, which, by contrast, also strongly reduced accumulation of *REP2*-derived 24-nt siRNAs (Fig. 4E). The levels of sRNAs derived from DCL1ci-interacting *TEs* were slightly but consistently reduced in *ago4* mutant tissues, suggesting that AGO4 loads and stabilizes these sRNAs, as is indeed the case of *REP2*-derived siRNAs (Fig. 4F). Northern analysis of AGO RIPs confirmed this interpretation (Fig. 4G) and also revealed strong loading in AGO9 but not in AGO6, despite both being closely related AGO4-clade members.

Taking into consideration the genetic requirements for sRNA production uncovered here at the DCL1ci-interacting *TEs*, we propose a hypothetical model whereby DCL1 might recognize discrete dsRNA regions within *TE* transcripts, perhaps upon scanning. DCL1 would then produce one or two direct cuts upon which *TE* transcripts would be converted by RDR2 into dsRNA eventually processed by DCL3 into 24-nt sRNAs. The nonrequirement for HYL1 in this process, unlike in miRNA biogenesis, could reflect that the *TE*-interacting DCL1 microprocessor is distinct from the miRNA processing complex. Alternatively, the precision granted by HYL1 to the maturation of discrete and sequence-invariable miRNA species during DCL1-mediated pri→pre→mature miRNA processing [77] might be superfluous for the suggested biogenesis of *TE*-derived siRNA populations.

Key RdDM pathway components are required for DCL1-dependent biogenesis of *TE*-derived siRNAs. Therefore, we inspected the cytosine methylation status of their 120 loci-of-origin, taking advantage of public bisulfite sequencing data available for most RNA silencing mutants of Arabidopsis [78]. High mCG, medium mCHG and low mCHH levels were observed over the 120 regions in WT replicates. As expected, mCHG and mCHH levels were reduced in most of the tested RdDM mutants albeit to different extents (Supplementary Fig. S4A). Unlike on conventional RdDM-targeted loci, however, the impact of *dcl3* was very mild on mCHH- and not visible on mCHG- levels. Inspecting bisulfite sequencing data from *dcl1, dcl234* and *dcl1234* mutant inflorescences also revealed that mCHG and mCHH levels on the 120 loci were both slightly but consistently increased in *dcl1* compared to WT. Similarly, the decreased methylation levels in *dcl234* were restored to WT in *dcl1234* (Supplementary Fig. S4A). Altogether, these results report a hitherto unknown group of *TE-*derived RNA substrates for DCL1. These fundamentally differ from pri-miRNAs because, at those loci, DCL1 promotes POLIV-RDR2-DCL3-AGO4/9-dependent and HYL1/POLV-independent sRNA accumulation that, counter-intuitively, antagonize RdDM via mechanisms that will require further investigations.

### Multiple feedback loop regulations on the *DCL1* mRNA

Transcripts from several *PCGs* were found to interact with DCL1ci (Fig. 3B), yet most only spawned low levels of sRNAs (Fig. 3I). In several cases, DCL1ci was found to interact prominently over 5′ and 3′UTR sequences. One such example is the 5′ UTR of the longest mRNA isoform of *DCL1* itself (Fig. 4H and Supplementary Fig. S4B). Interestingly, it has been shown that the mRNA from *DGCR8*, involved in pri-miRNA processing in mammals, contains conserved secondary structure enabling its posttranscriptional control by the Dicer-like RNase III Drosha [79, 80]. DCL1ci interaction with the *DCL1* mRNA was located on the 5′UTR, leading us to explore this specific region across Brassicaceae. We retrieved the corresponding genomic sequences of *DCL1* homologues and compared them by multiple alignment (see the ‘Materials and methods’ section). One of the best conserved regions identified corresponded to the 5′UTR region (∼50 nt) specifically enriched by DCL1ci RIP in *A. thaliana* (Supplementary Fig. S4C). Applying Gapped Local Alignment of Motifs (Glam2 [81]) we identified only two small regions of ∼7-nt (Supplementary Fig. S4D), suggesting that the conserved detected region across Brassicaceae cannot be solely explained by transcription factor binding. Hence, we explored both local and global secondary structure-folding over the *DCL1* 5′UTR sequence as well as its possible conservation in Brassicaceae. Scanning the Arabidopsis *DCL1* 5′UTR using scanFold identified three significant (p-score < 0.01) short (∼150 nt) stem-loops of which one overlaps the peak of the *A. thaliana* DCL1ci RIP-seq (Supplementary Fig. S4E). Secondary structure conservation analysis detected this stem-loop in Arabidopsis’ close relatives (Supplementary Fig. S4F) but not in more evolutionary distant Brassicaceae. Comparing the sequence and structure of the full-length *DCL1* 5′UTRs among all inspected Brassicaceae identified a longer secondary structure with a highly conserved stem but more divergent loop region (Fig. 4H and I, and Supplementary Fig. S4G). One side of the conserved stem corresponds to the sequence significantly enriched in the DCL1ci-RIP, while the other corresponds to a window that, while also significantly enriched, did not pass our minimal-reads cutoff (Fig. 4I).

Both the smaller and/or longer secondary structure(s) could therefore explain the DCL1ci-IP enrichment over the *DCL1* 5′UTR. These could potentially allow DCL1 feedback-regulation via the control of its own mRNA. No sRNA reads could be mapped onto the *A. thaliana* DCL1ci-interacting region, however. Thus, the suggested auto-regulation might proceed via single-cleavage of the *DCL1* mRNA or by mere DCL1 binding and recruitment of other factors to perhaps regulate *DCL1* translation. Two negative-feedback loops are already known to control the *DCL1* mRNA in Arabidopsis [82]. DCL1-dependent miR162 targets *DCL1* for cleavage at a complementary site formed by the splicing-dependent connection of exon 12 to exon 13 [83]. Additionally, intron 14 of *DCL1* forms a hairpin structure generating the miR838 mirtron. DCL1-mediated processing of the *DCL1* pre-mRNA releases pre-miR838, yielding nonproductive fragments of *DCL1* transcripts [31]. These already existing DCL1-dependent mechanisms make it difficult to specifically assess, in the *dcl1* mutant background, the feedback-regulatory potential of the newly identified 5′ UTR-binding sites. Engineering miR162-resistant cDNA bearing the authentic or modified *DCL1* 5′UTR sequence/structure will help addressing this question. Regardless, given the central role for DCL1 in miRNA biogenesis alone, the existence of multiple control points would not be surprising, including via the hitherto unknown and specific interaction reported here between DCL1 and its own transcript.

## Conclusion

Our transcriptome-wide RIP-Seq analysis of DCL1ci in Arabidopsis inflorescences provides several key insights into the breadth and mechanics of DCL1 substrates’ recognition well-beyond classical pri-/pre-miRNAs. First, by stabilizing protein–substrate complexes, DCL1ci enabled the direct capture of over 30% of annotated pri-miRNAs, with a pronounced enrichment for evolutionarily ancient loci that rely exclusively on DCL1 for precise processing. This validates the approach’s sensitivity and establishes DCL1ci-RIP as a powerful method to survey miRNA precursors in planta. Noteworthy, we also found that WT plants transformed with *pDCL1:DCL1ci* exhibit a *dcl1-*like phenotype (Supplementary Fig. S4H). Thus, catalytically inactive DCL1ci likely competes with endogenous DCL1 for substrate binding when expressed in WT plants, thereby interfering with normal miRNA processing. Conceivably, tissue- or cell-specific expression of DCL1ci in WT plants might enable an exploration of miRNA deficiency at cellular resolution without the widespread developmental complications of the *dcl1* mutant background. Second, the profiling of RIP-Seq signal distributions revealed that DCL1 engages not only the imperfect stem-loop regions of pre-miRNAs, but also flanking single-stranded segments. These observations support a model in which DCL1 scans along the primary transcript potentially via transient interactions with ssRNA regions to locate and process the miRNA-encoding hairpin. The variable peak enrichments on guide versus passenger strands could reflect mechanistic diversity in processing modes among different *MIRNA* families. Third, DCL1ci-RIP provides the first quantitative discrimination among paralogous *MIRNA* loci. In numerous multi-member families, only a subset of paralogs exhibited significant DCL1ci binding in our tissue-specific assay, highlighting differential transcriptional activity and/or structural features that dictate distinctive precursor engagement. This capability to resolve family member usage promises to refine models of mRNA-based gene regulation across development and environmental conditions. Fourth, our genome-wide survey uncovered hundreds of additional DCL1-interacting loci, including protein-coding genes, *TEs*, and intergenic regions. A substantial fraction of these sites lack canonical hairpin structures, suggesting that DCL1’s substrate specificity is more promiscuous than previously appreciated and may involve diverse RNA architectures. Functional assays confirmed that DCL1 contributes to siRNA production from endogenous *IRs* (e.g. *IR71*) and promotes non-miRNA-like sRNA biogenesis from transposon transcripts in a manner genetically distinct from the canonical RdDM pathway. Collectively, these findings implicate DCL1 in cross-talk among multiple silencing branches, potentially modulating gene and transposon expression via coordinated small RNA outputs. Finally, the identification of a binding site in a partially conserved stem-loop motif within the 5′ UTR of the *DCL1* transcript itself hints at an auto-regulatory feedback mechanism. DCL1 binding to its own mRNA may modulate translation or transcript stability in concert with the established miR162- and miR838-mediated loops, thereby fine-tuning *DCL1* cellular levels. In summary, DCL1ci-RIP extends our understanding of plant DCL1 functions beyond miRNA biogenesis. It reveals substrate flexibility, unveils novel sRNA sources, and exposes regulatory feedback intricacies. This approach also lays the foundation for generic, cell/tissue-type- or condition-specific maps of other DCL activities, including in nonplant organisms such as metazoans.

## Supplementary Material

gkaf1434_Supplemental_Files

## Data Availability

Data are available on the NCBI Gene Expression Omnibus (GEO) under the accession number GSE192355.

## Notes

Present address: Department of Environmental Systems Sciences, Swiss Federal Institute of Technology (ETH), Zürich, 8092, Switzerland
