# Correction to ‘Transcriptome-wide analysis of Arabidopsis DICER-LIKE1 RNA substrates’

**DOI:** 10.1093/nar/gkag872

**Published:** 2026-10-15

This is a correction to: Nicolas G Bologna, Alexis Sarazin, Gregory Schott, Antoine Bouet, Natalia P Achkar, Belen Moro, Florence Jay, Uciel Chorostecki, Emanuel A Devers, Olivier Voinnet, Transcriptome-wide analysis of Arabidopsis DICER-LIKE1 RNA substrates, *Nucleic Acids Research*, Volume 54, Issue 3, 10 February 2026, gkaf1434, https://doi.org/10.1093/nar/gkaf1434

Distinct catalytic glutamate residues were inadvertently misassigned, leading to the erroneous reporting of the mutations as E1378Q and E1597Q instead of the correct E1508Q and E1697Q. This has been corrected in section **Results and discussion**, under heading **Catalytically inactive DCL1 interacts with the microprocessor component HYL1 and stabilizes co-immunoprecipitated pri-miRNA**. The correction is made to the text in the first sentence to read: “(E1508Q and E1697Q)” instead of: “(E1378Q and E1597Q)”

The error also affects Supplementary Figure S1B which should read:

**Figure:**
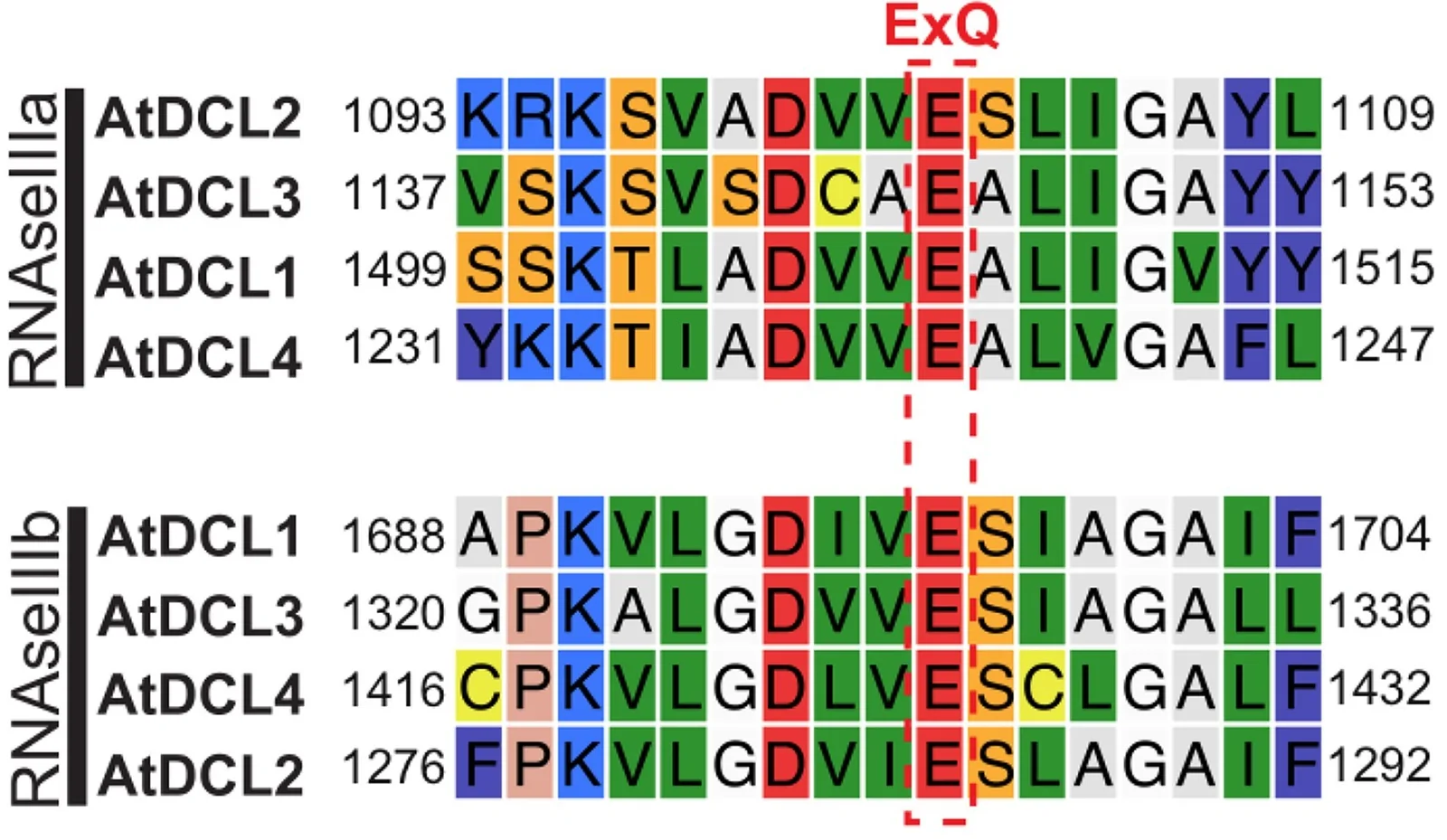


instead of:

**Figure:**
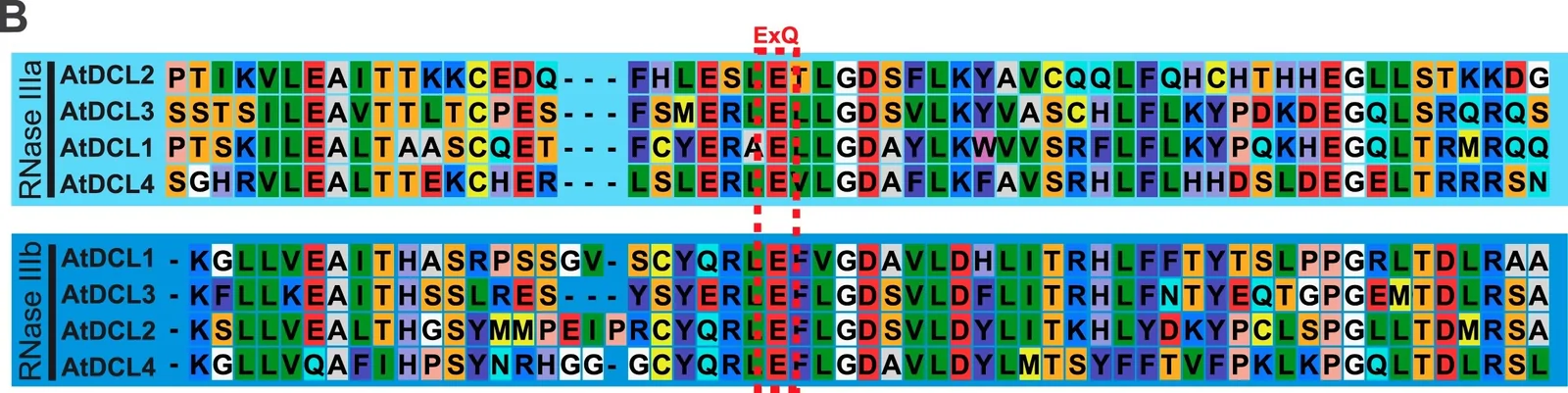


This has also been corrected in the respective **Supplementary data** file.

While the mistake does not affect the results or conclusions presented in the manuscript, this correction is to ensure clarity and accuracy, particularly for researchers who may wish to reproduce these exact mutations in Arabidopsis', other plant species', or metazoan's DCLs.

