# Supplementary material for: Transcriptome-wide analysis of Arabidopsis DICER-LIKE1 RNA substrates
Source: Nucleic Acids Res. 2026 Jan 22;54(3):gkaf1434. doi: 10.1093/nar/gkaf1434 (PMC12825306; doi:10.1093/nar/gkaf1434)
Supplement: gkaf1434_Supplemental_Files [file gkaf1434_supplemental_files.zip › Revised Supp Material_Bologna et al 17.11.pdf]

SUPPLEMENTARY FIGURES

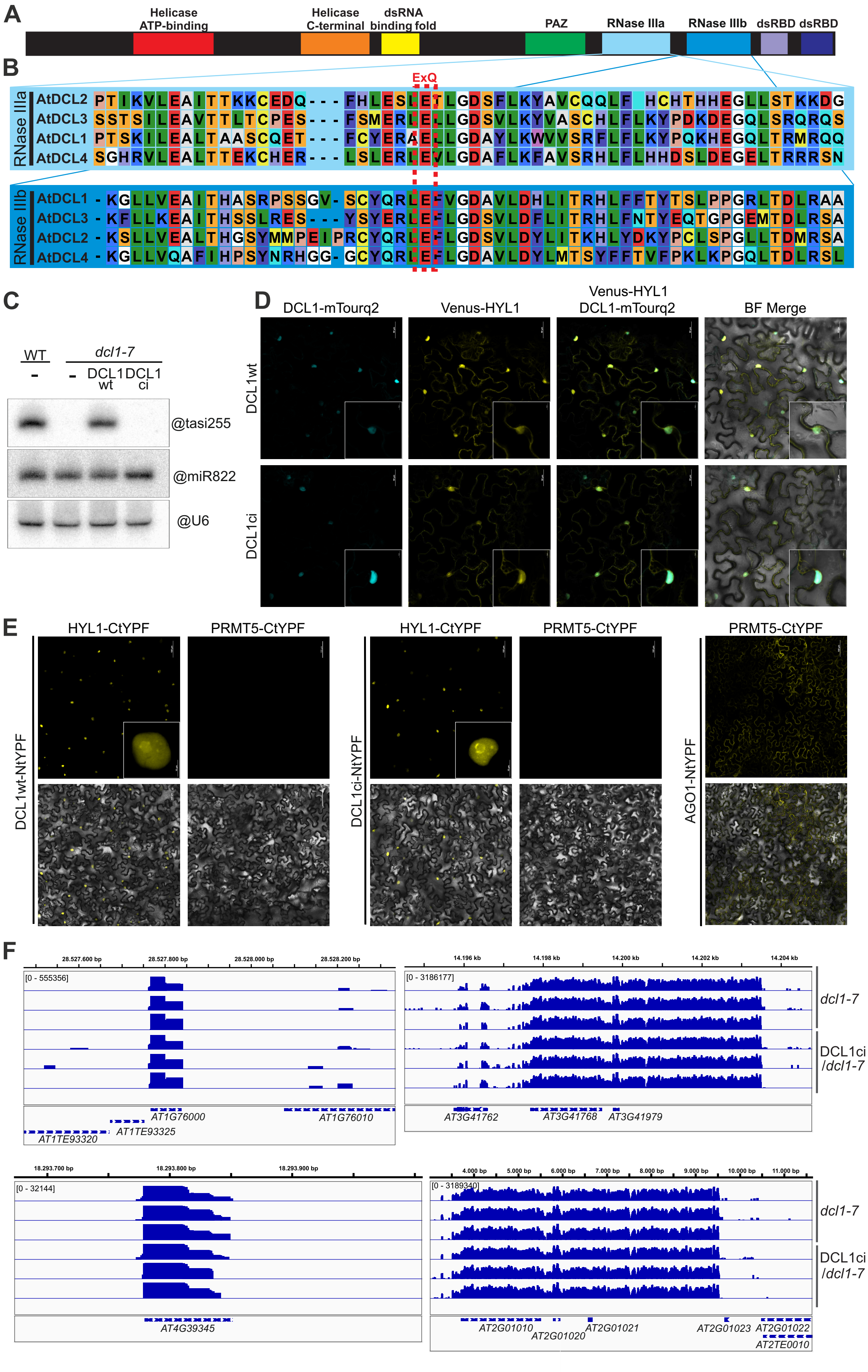

Supplementary Figure S1

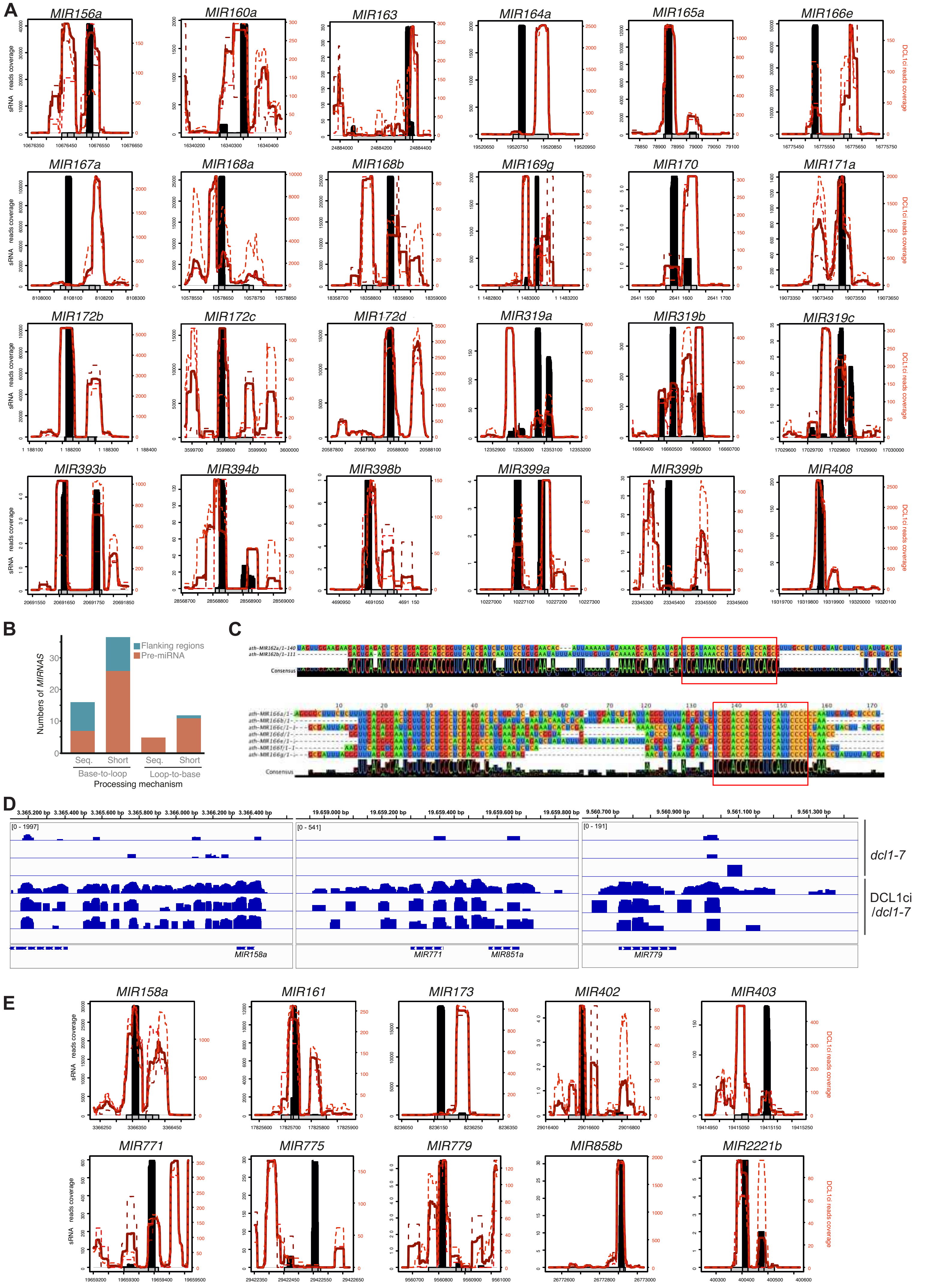

Supplementary Figure S2

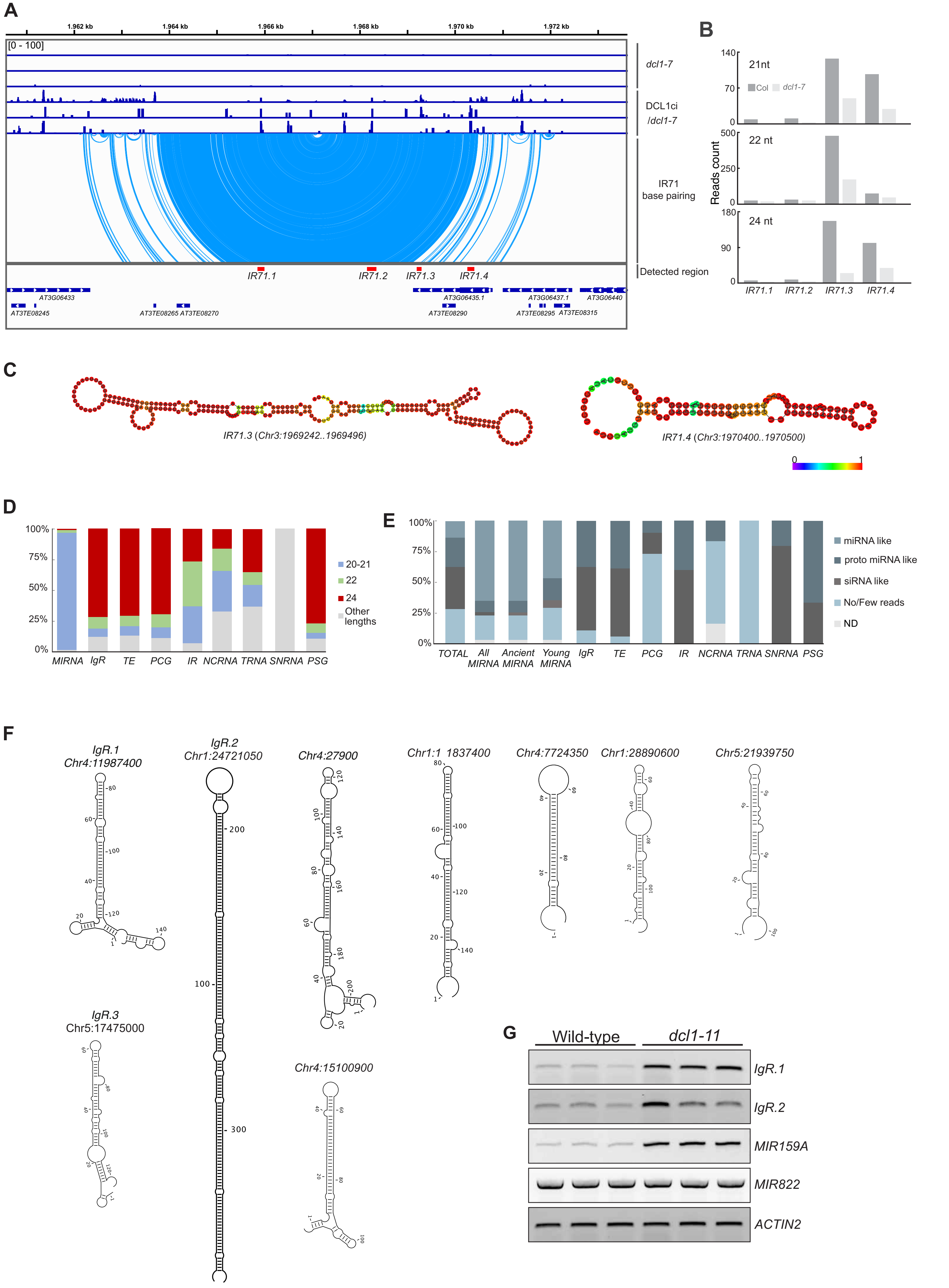

Supplementary Figure S3

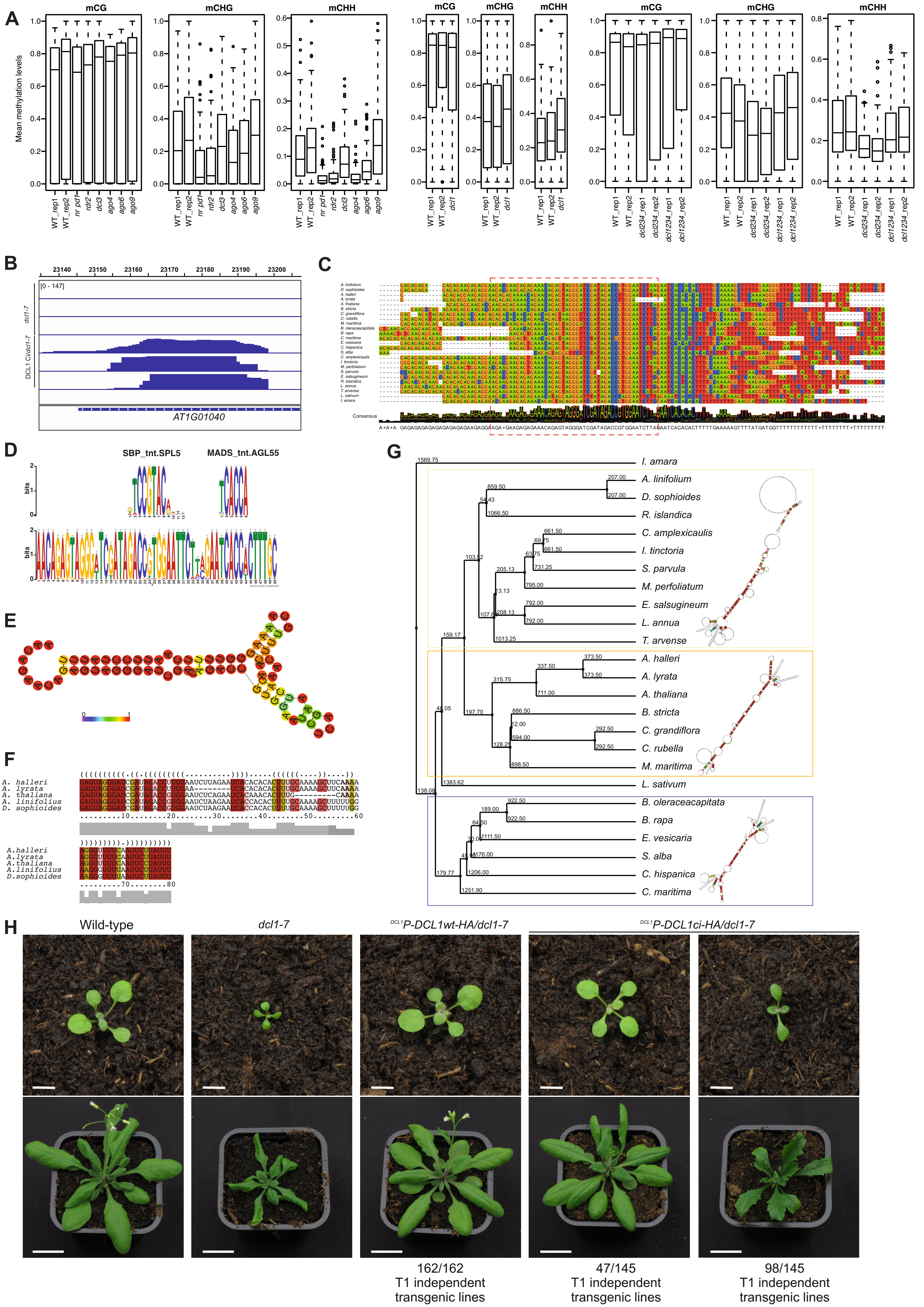

Supplementary Figure S4

SUPPLEMENTARY FIGURES LEGENDS

Supplementary Figure S1. (**A**) Schematic diagram of domains in DICER-LIKE proteins. **(B)** Amino acid alignment of two RNase III domains from DCL1, DCL2, DCL3, and DCL4 proteins. (**C**)**.** Northern blot analysis of tasi255 and miR822 levels in ^{DCL1}P-DCL1wt-HA/*dcl1-7*, ^{DCL1}P-DCL1ci-HA/*dcl1-7,* and Col-0 and *dcl1-7* control plants. U6 is used as a loading control. (**D**) Transient BiFC assays in *N.benthamiana* leaves used to test interaction between DCL1ci (*35S:DCL1ci-NtYFP*) and HYL1 (*35S:HYL1-CtYFP*). PRMT5 (*35S:PRMT5-NtYFP*) was used as a negative control, while DCL1wt (*35S:DCL1wt-NtYFP*) and AGO1 (*35S:NtYFP-AGO1*) were used as positive controls(72). (**E**) Transient expression assays in *N.benthamiana* leaves used to test colocalization between DCL1ci (*35S:DCL1ci-mTourq2*) and HYL1 (*35S:Venus-HYL1*). DCL1wt (*35S:DCL1wt-mTourq2*) was used as a positive control. (**F**) IGV visualizations of read coverage of DCL1P-DCL1ci-HA/dcl1-7 and dcl1-7 control plants (three replicates for each) over two tRNA genes (AT1G7600 and AT4G39345; left) and two regions containing rRNA genes (right). Visualizations are done using the Log Group Autoscale options, maximum coverage is indicated on the first track.

Supplementary Figure S2. (**A**) Coverage of sRNA annotations (black histogram) versus actual RNA seq reads from DCL1ci-RIP (red line) over several detected evolutionarily conserved precursors. The plain thick red lines correspond to the average of three replicates represented with dashed thin red lines. Light and dark gray rectangles represent primary transcripts and mature 5p/3p miRNA annotations, respectively. (**B**) Most enriched DCL1ci-RIP peak for each pri-miRNAs were classified as either within the pre-miRNA or in the flanking regions (±100-nt relative) and compared across processing mechanisms: short or sequential base-to-loop, or short or sequential loop-to-base. (**C**) Full multiple-alignment of stem-loop sequences from *MIR162* and *MIR166* family members. Red squares indicate mature miRNAs. (**D**) IGV visualizations of read coverage of DCL1P-DCL1ci-HA/dcl1-7 and dcl1-7 control plants (three replicates for each) over four young miRNA genes (*MIR158a*, *MIR771*, *MIR779*, and *MIR851a*). Visualizations are done using the Log Group Autoscale options, maximum coverages are indicated on the first track. (**E**) Same as S2A for several evolutionary young miRNA primary transcript annotations.

Supplementary Figure S3. (**A**) IGV visualization of reads coverage of DCL1P-DCL1ci-HA/dcl1-7 and dcl1-7 control plants (three replicates for each) over the long inverted-repeat IR71. Cyan arcs indicate the base pairing predicted using RNAfold. Red rectangles correspond to the regions detected as significantly enriched over IR71. Annotations (genes and transposons) are shown at the bottom. (**B**) sRNA reads from wild-type and *dcl1-7* Arabidopsis plants generated from the four IR71 regions detected by DCL1ci RIP. (**C**) Local secondary structure predicted with RNAfold for the regions IR71.3 and IR71.4. Genomic positions of the sequences used for the prediction are indicated. (**D**) sRNA length proportion within each annotation-types for regions enriched by DCL1ci-RIP in Figure 3A-C. (**E**) Categorization of the sRNA patterns associated with DCL1ci-binding sites.sRNA length proportion given all the different annotation types of regions detected with DCL1 RIP. (**G**) sqRT-PCR of transcript from hairpins represented in B in three independent biological replicates of *dcl1-11* or WT inflorescences. DCL1-dependent *MIR159A*, DCL4-dependent *MIR822* and *ACTIN2* are used as positive, negative, and equal loading control, respectively.

Supplementary Figure S4. (**A)** Boxplot representation of the average cytosine methylation level of the 120 regions overlapping transposable element sequences detected by DCL1ci RIP in various RdDM mutants as well as *dcl1*, *dcl234*, *dcl1234* and their respective WT control in the three cytosine contexts (CG, CHG and CHH). (**B**) IGV visualization of reads coverage of DCL1P-DCL1ci-HA/*dcl1-7* and *dcl1-7* control plants (three replicates for each) over the beginning of DCL1 5’ UTR sequence. This is a zoom-in from Figure 4H. (**C**) Multiple alignment of the beginning of DCL1 5’ UTR sequence in several Brassicaceae. Dashed red box corresponds to the peak observed in S4B. (**D**) Weblogo visualization of the 50bp motif detected by Glam2 as conserved in Brassicaceae DCL1 5’ UTR sequence (bottom) with the example of two transcription factor binding sites overlapping part of it (top). (**E**) Local secondary structure detected by scanFold in Arabidopsis thaliana DCL1 5’ UTR sequence. (**F**) Representation of the conservation of the RNA secondary structure from S4E in species closely related to Arabidopsis thaliana as detected using LocARNA. (**G**) Cladogram based on the Average distance of DCL1 5’ UTR sequence from Brassicaceae calculated after multiple alignment with T-Coffee. Consensus secondary structure predicted by LocARNA/RNAalifold for several clades are included. Base

pairs are colored such that hue shows sequence conservation and saturation shows structural conservation. (**H**) Phenotypes of wild-type, *dcl1-7,* and wild-type plants expressing wild-type (wt) or catalytically inactive (ci) genomic DCL1 under the *DCL1* endogenous promoter fused to Human influenza hemagglutinin (HA). Among the DCL1wt-expressing plants, 100% (162/162) displayed a wild-type phenotype. In contrast, only ~30% (47/145) of DCL1ci-expressing plants showed a wild-type phenotype, while the remaining 70% (98/145). Scale bars: 0,5 cm (top) and 2 cm (bottom).

SUPPLEMENTARY TABLES LEGENDS

Supplementary Table S1: Raw reads count from DCL1ci IP for annotated pri-miRNAs, tRNA, and rRNA. Results of DESeq2 normalization and differential expression are also included.

Supplementary Table S2: sRNA sequencing data from WT and *dcl1-7* inflorescences for pri-miRNAs loci (Jeong et al. 2013). The table include the number of 20-21-nt, 22-nt and 24-nt long reads in WT library, the reads count in both WT and *dcl1-7* libraries normalized by library size and number of genomic positions. The log fold change of those values is also included as well as the log2foldChange and p-value from Table S1.

Supplementary Table S3: Regions detected as significantly enriched in ^{DCL1}P-DCL1ci-HA/*dcl1-7* RIP in comparison *dcl1-7* RIP controls. The table include genomic positions of the merged enriched genomic windows (see methods), annotation of the region as well as raw and normalized reads count in the six RIP-seq libraries.

Supplementary Table S4: Pre-miRNA-like secondary structures detected in genomic positions identified in DCL1ci IP. Sequence and predicted structure are indicated in dot bracket notation are provided.

Supplementary Table S5: Quantitative and qualitative characterization of WT sRNA accumulating over the regions enriched in DCL1ci-IP. Information include number of reads (nbseq), number of distinct reads (nbdiffseq), number of reads depending of their length (20-21nt, 22nt, 24nt, other = lg2021,lg22,lg24 and OtherLength), their strand (nbW, nbC = number of reads on plus or minus strand), the minimum, mean and maximum number of reads for the distinct sequences corresponding to the regions

(minCount, meanCount, mxCount) and minimum, mean and maximum number of genomic position for the distinct sequences corresponding to the regions. Those informations were used to classify the sRNA pattern in different Categories (CAT) also provided.

Supplementary Table S6: AGO1 loaded sRNA reads derived from 3 IgR regions enriched in DCL1ci-IP and predicted to generate a secondary structure. For each IgR, most abundant sRNA reads from 2 replicates of AGO1-IP libraries are included as well as their target prediction, with psRNAtarget, over the selected candidate genes. Only prediction with an Expect score lower or equal to 2 are included.

Supplementary Table S7: Small RNA reads in WT and *dcl1-7* inflorescences over DCL1ci-IP enriched regions regions. For each DCL1ci-IP enriched regions, the table provide raw and normalized reads count (normalization by library size and number of genomic position) for 20-21-nt, 22-nt and 24-nt long reads obtain from WT and *dcl1-7* public libraries. Similar values are also provided for all A.th annotations (TAIR10). Normalized value from 24-nt long reads from TE and TE regions were used for Figure 4C.

Supplementary Table S8: Oligos, probes and antibodies.
