# Supplementary material for: Transcriptome-wide analysis of Arabidopsis DICER-LIKE1 RNA substrates
Source: Nucleic Acids Res. 2026 Jan 22;54(3):gkaf1434. doi: 10.1093/nar/gkaf1434 (PMC12825306; doi:10.1093/nar/gkaf1434)

Final Figure 1C

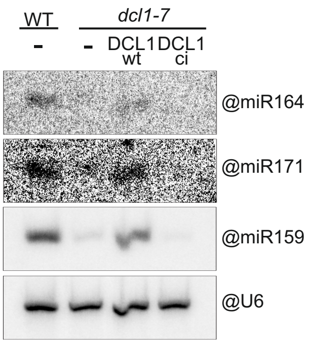

Raw data for Figure 1C

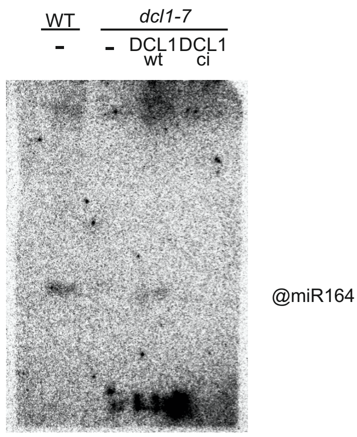

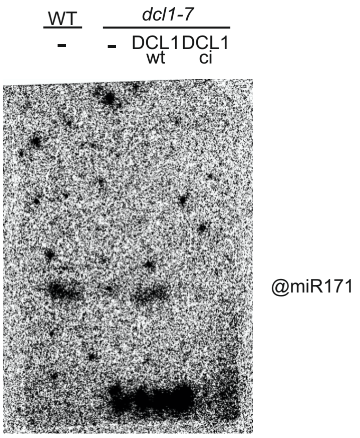

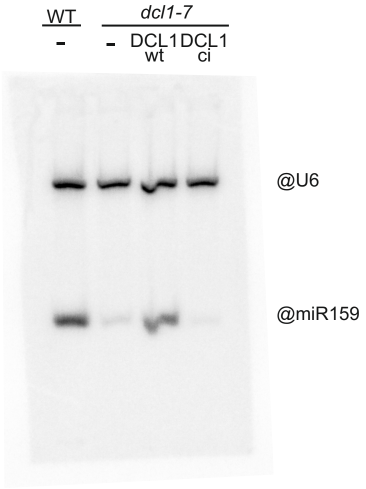

The membrane was stripped and re-probed multiple times

Final Figure 1D

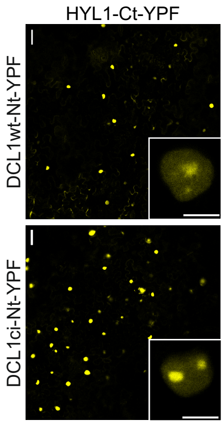

Raw data for Figure 1D

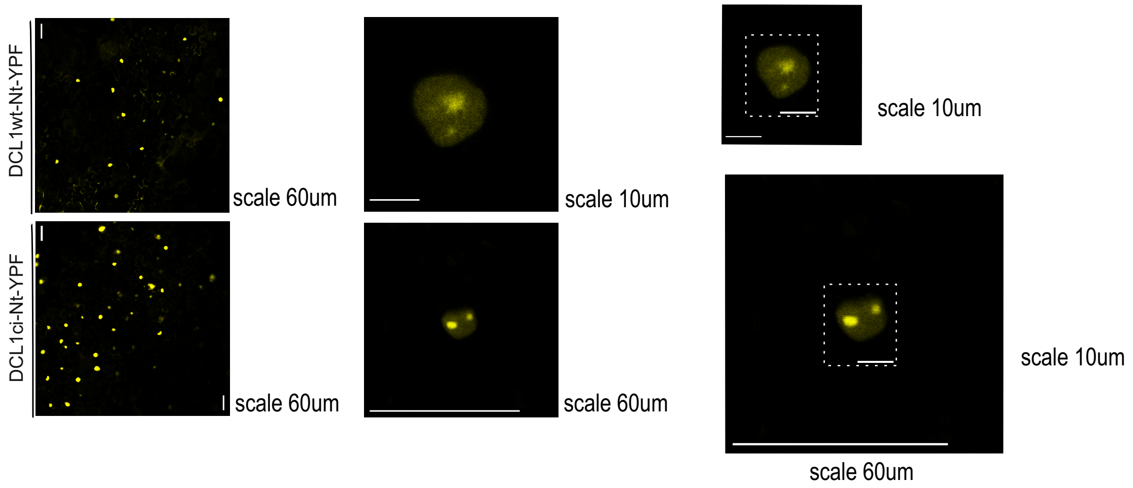

Final Figure 1E

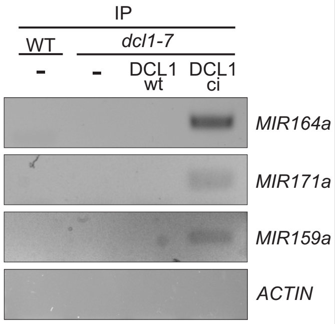

Raw data for Figure 1E

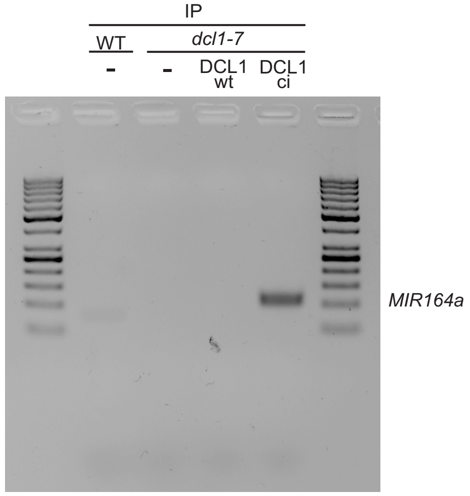

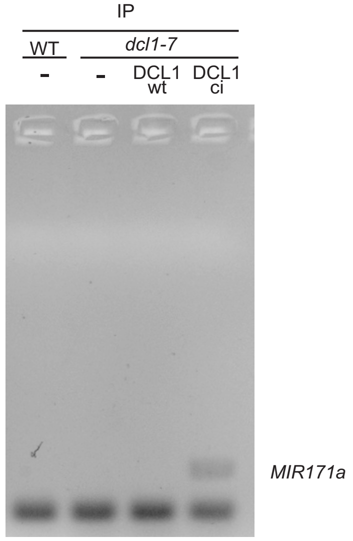

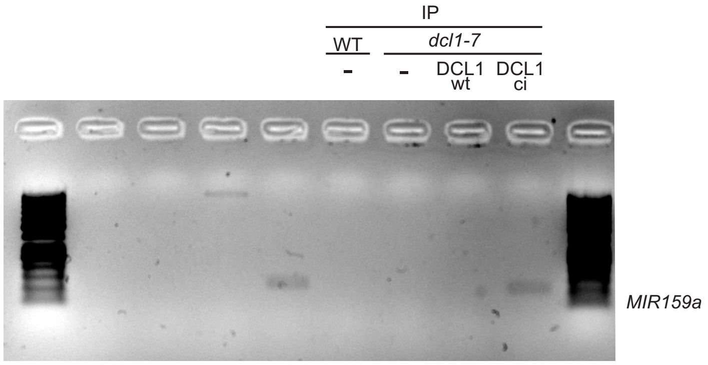

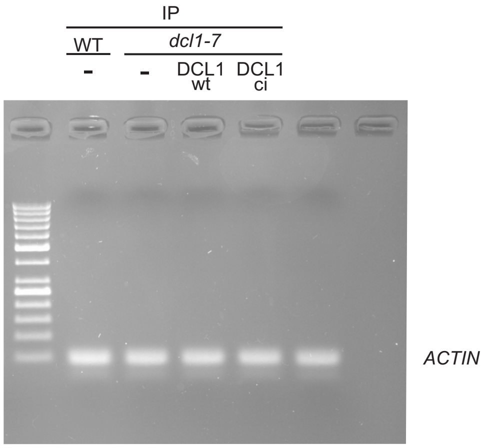

Final Figure 2K

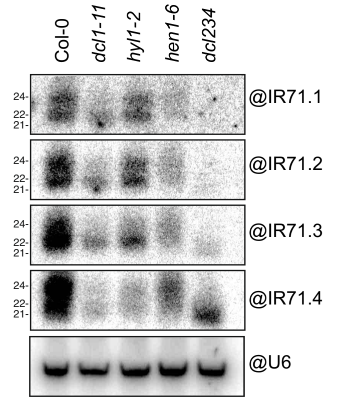

Raw data for Figure 2K

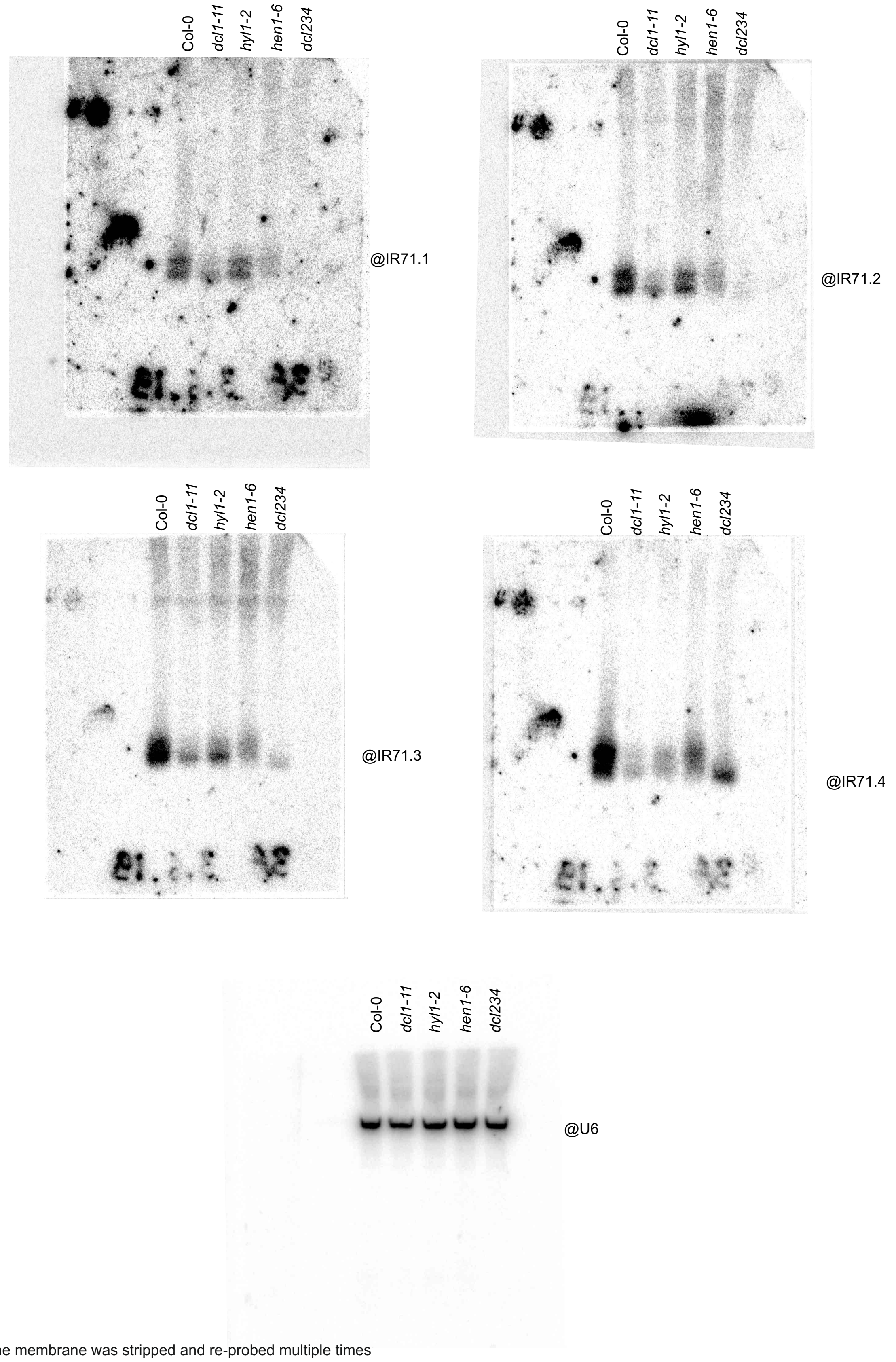

The membrane was stripped and re-probed multiple times

Final Figure 4D

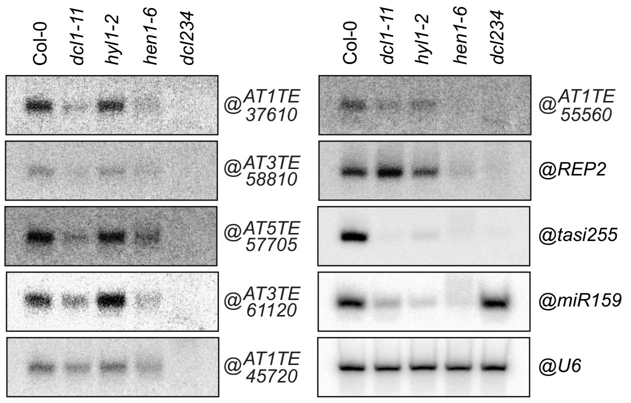

Raw data for Figure 4D

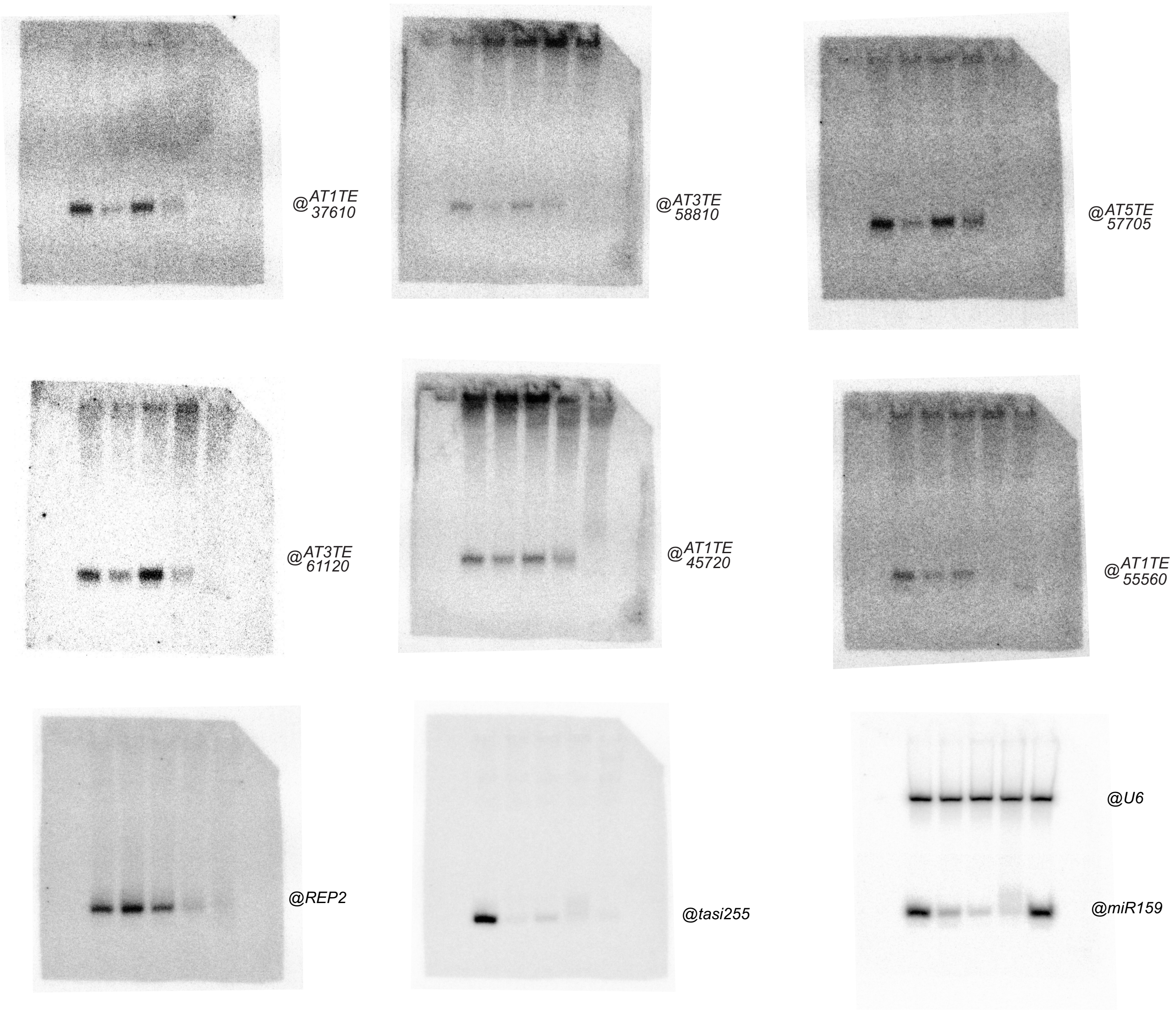

The membrane was stripped and re-probed multiple times

Final Figure 4E

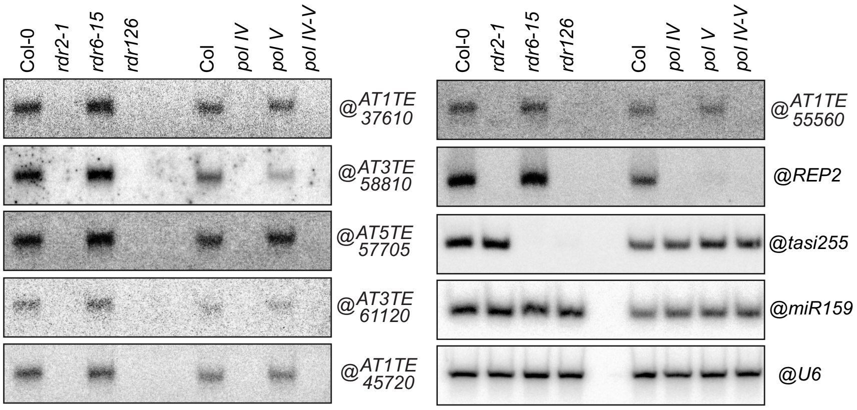

Raw data for Figure 4E

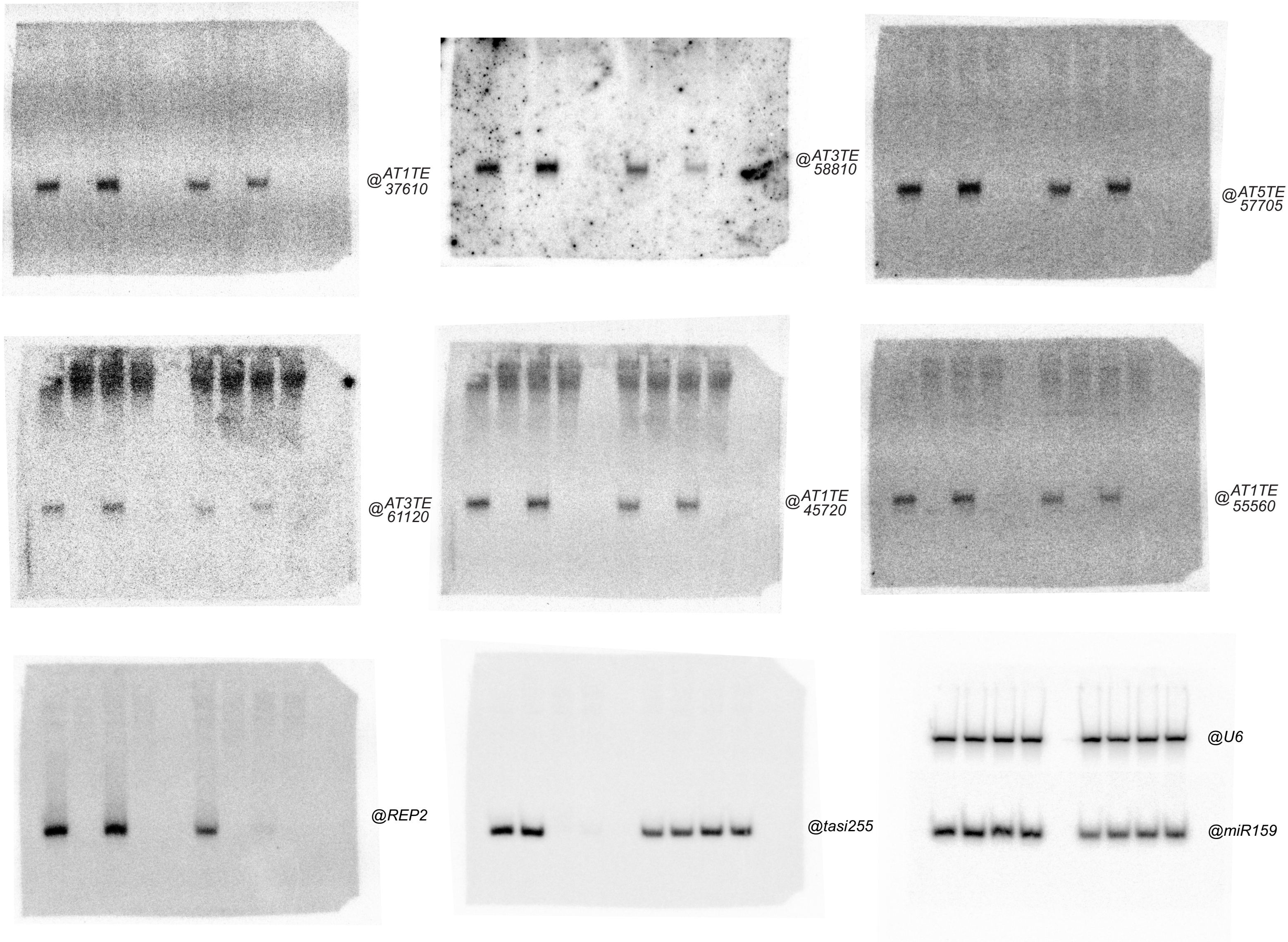

The membrane was stripped and re-probed multiple times

Final Figure 4F

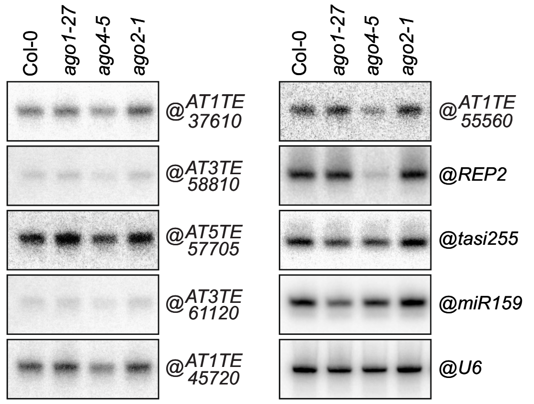

Raw data for Figure 4F

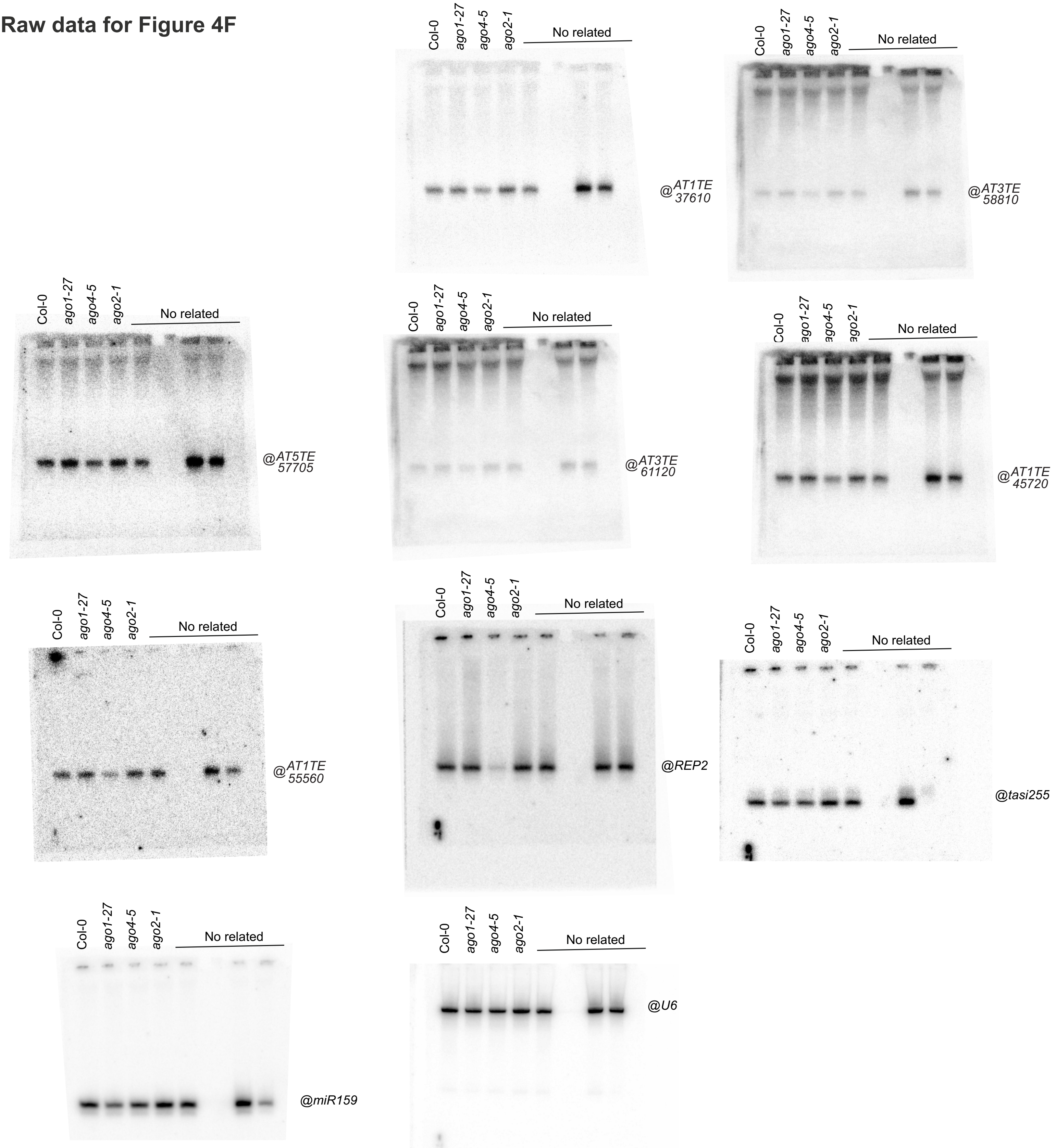

The membrane was stripped and re-probed multiple times

Final Figure 4G - Northern blot

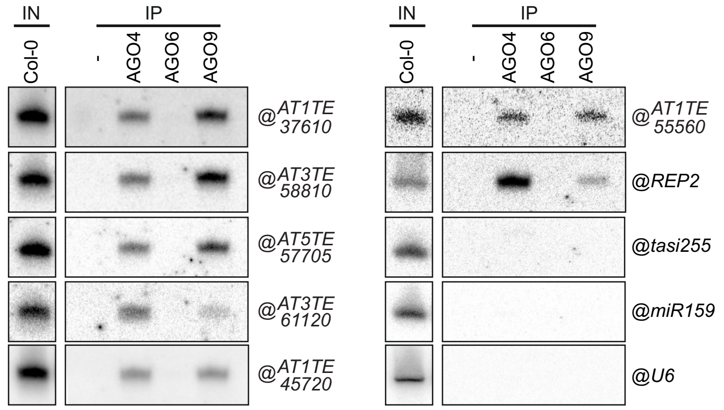

Raw data for Figure 4G - Northern blot

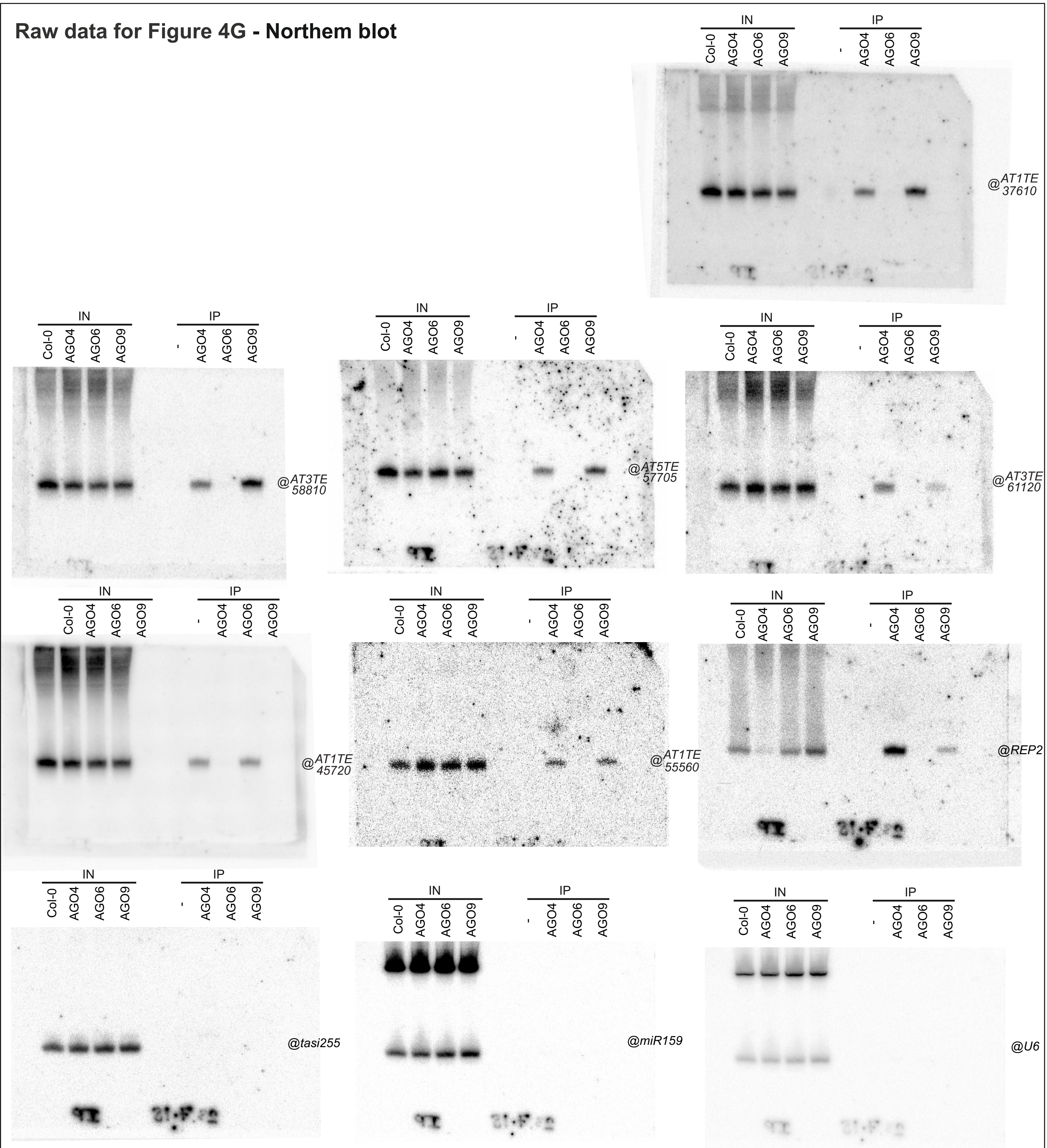

The membrane was stripped and re-probed multiple times

Final Figure 4G - Western blot (cont.)

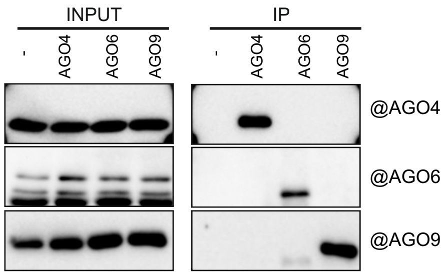

Raw data for Figure 4G - Western blot (cont.)

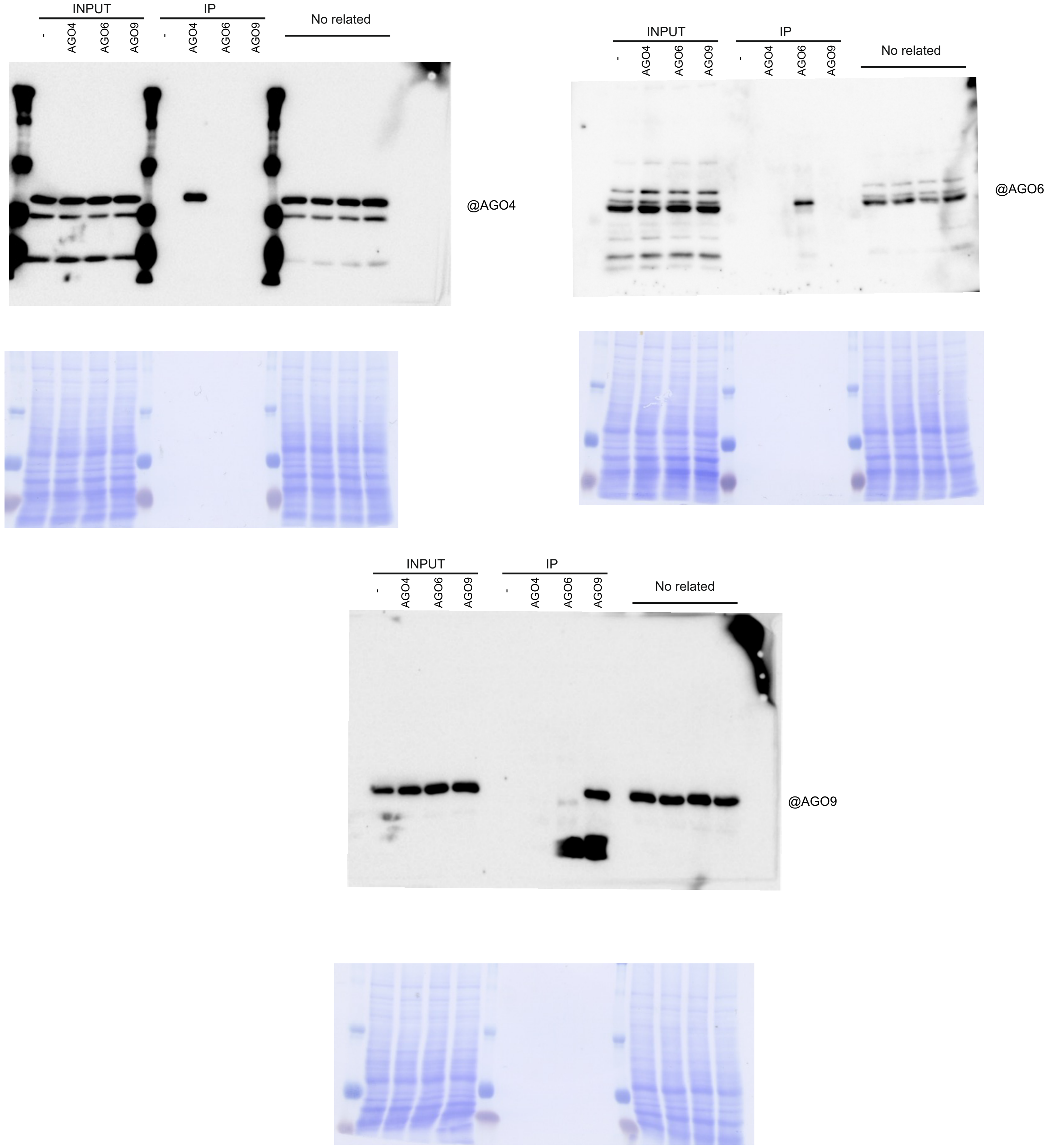

Final Figure Supplementary S1C

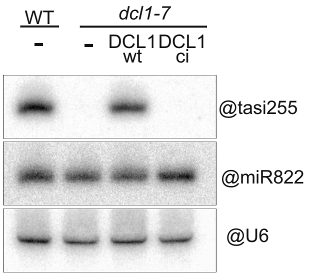

Raw data for Supplementary Figure S1C

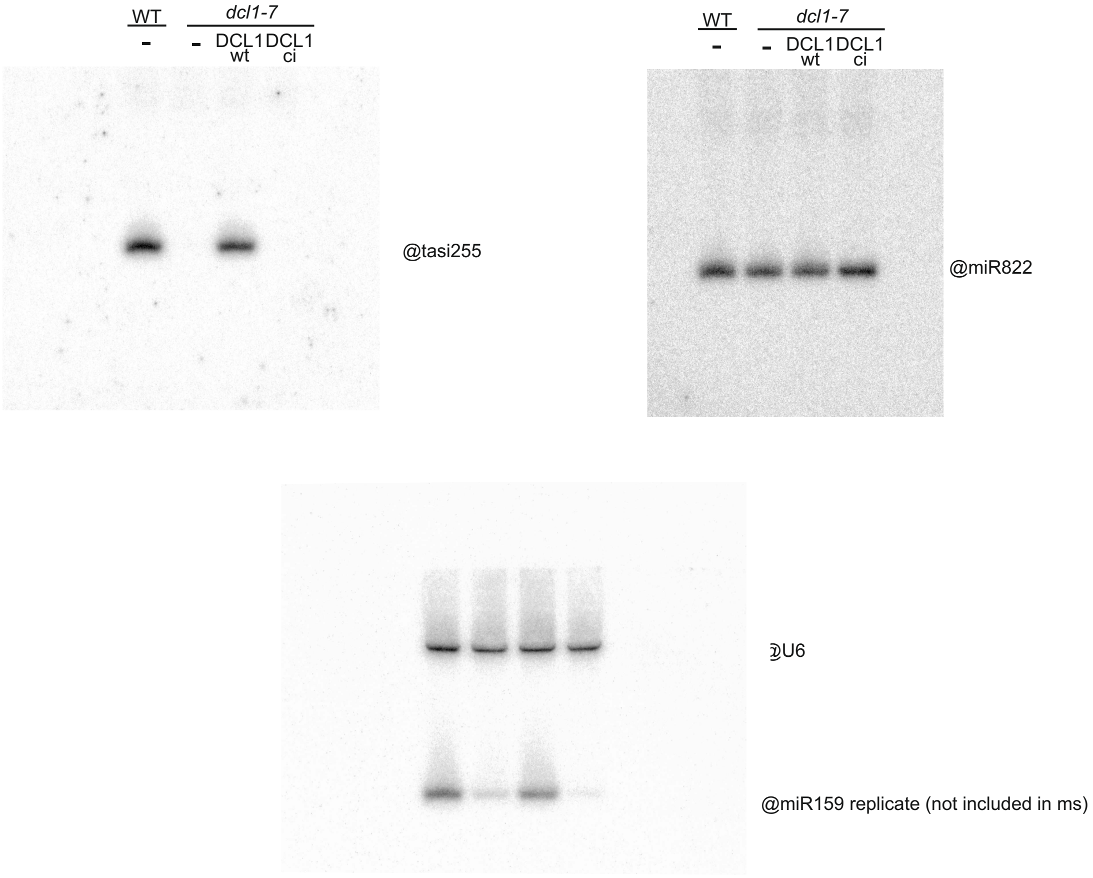

The membrane was stripped and re-probed multiple times

Final Figure
Supplementary S3G

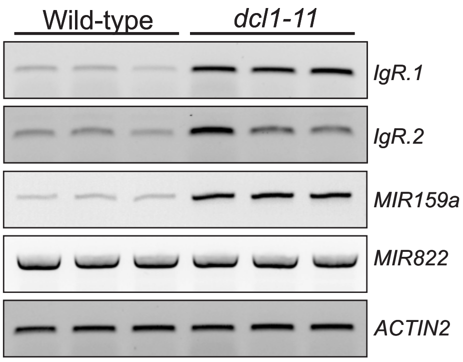

Raw data for Supplementary Figure S3G

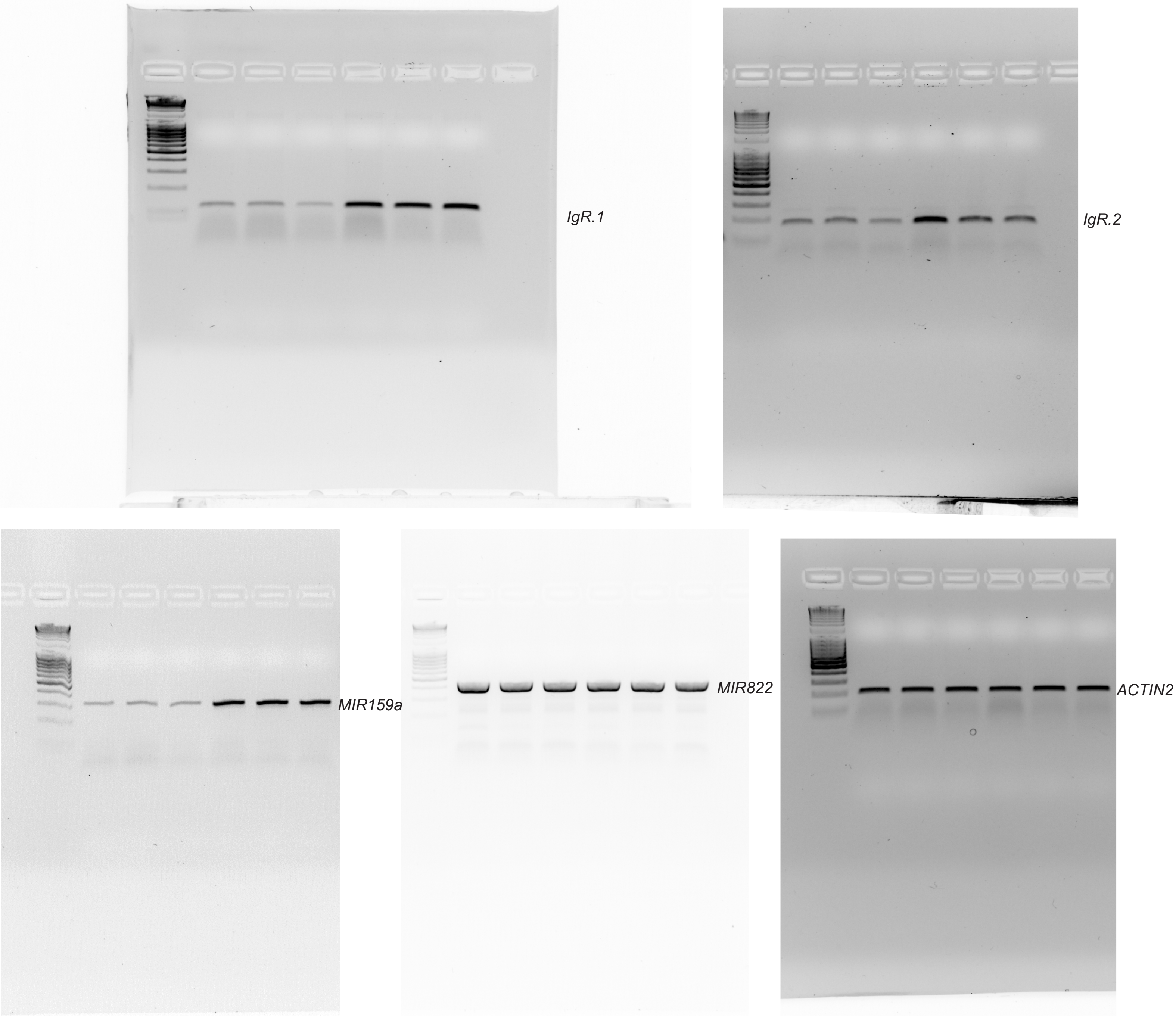

Supplement: gkaf1434_Supplemental_Files [file gkaf1434_supplemental_files.zip › Bologna et al_Raw data_ 25.11.pdf]
